# Genetic and Biochemical Characterization of the MinC-FtsZ Interaction in *Bacillus subtilis*

**DOI:** 10.1371/journal.pone.0060690

**Published:** 2013-04-05

**Authors:** Valdir Blasios, Alexandre W. Bisson-Filho, Patricia Castellen, Maria Luiza C. Nogueira, Jefferson Bettini, Rodrigo V. Portugal, Ana Carolina M. Zeri, Frederico J. Gueiros-Filho

**Affiliations:** 1 Departamento de Bioquímica, Instituto de Química, Universidade de São Paulo, São Paulo, Brasil; 2 Brazilian Biosciences National Laboratory (LNBio), Centro Nacional de Pesquisas em Energia e Materiais (CNPEM), Campinas, Brasil; 3 Nanotechnology National Laboratory (LNNano), Centro Nacional de Pesquisas em Energia e Materiais (CNPEM), Campinas, Brasil; Institut Pasteur Paris, France

## Abstract

Cell division in bacteria is regulated by proteins that interact with FtsZ and modulate its ability to polymerize into the Z ring structure. The best studied of these regulators is MinC, an inhibitor of FtsZ polymerization that plays a crucial role in the spatial control of Z ring formation. Recent work established that *E. coli* MinC interacts with two regions of FtsZ, the bottom face of the H10 helix and the extreme C-terminal peptide (CTP). Here we determined the binding site for MinC on *Bacillus subtilis* FtsZ. Selection of a library of FtsZ mutants for survival in the presence of Min overexpression resulted in the isolation of 13 Min-resistant mutants. Most of the substitutions that gave rise to Min resistance clustered around the H9 and H10 helices in the C-terminal domain of FtsZ. In addition, a mutation in the CTP of *B. subtilis* FtsZ also produced MinC resistance. Biochemical characterization of some of the mutant proteins showed that they exhibited normal polymerization properties but reduced interaction with MinC, as expected for binding site mutations. Thus, our study shows that the overall architecture of the MinC-FtsZ interaction is conserved in *E. coli* and *B. subtilis*. Nevertheless, there was a clear difference in the mutations that conferred Min resistance, with those in *B. subtilis* FtsZ pointing to the side of the molecule rather than to its polymerization interface. This observation suggests that the mechanism of Z ring inhibition by MinC differs in both species.

## Introduction

Cell division in bacteria is executed by a contractile protein apparatus that contains about twenty different components and which is commonly known as the divisome [1]–[3]. The divisome is an elaborate molecular machine that promotes the coordinated invagination of the bacterial cytoplasmic membrane and cell wall (plus the outer membrane, in the case of Gram-negative bacteria) to create the division septum. Assembly of the divisome from its isolated parts is orchestrated by FtsZ, the ubiquitous tubulin-like protein of bacteria [4]. FtsZ is a cytoskeletal protein that self-associates into a supramolecular structure with the shape of a ring [5], [6]. Once formed, this FtsZ (or Z) ring is capable of recruiting the other components of the divisome to the future division site. Thus, the Z ring functions as a seed and structural organizer of the divisome.

A remarkable feature of bacterial division is that it occurs with great spatial precision and in perfect coordination with DNA replication and segregation. Controlling where and when the divisome will assemble occurs primarily at the level of Z ring formation. There are several proteins that interact directly with FtsZ and either help or inhibit its self-assembly into the Z ring [7]. These proteins, which are often referred to as “FtsZ modulators”, are key players in the regulation of division.

The best studied modulator of Z ring formation is the Min system [8], [9]. The Min system represents a site-specific inhibitor of FtsZ polymerization that prevents re-initiation of division immediately after a round of division has finished [10], [11]. In the absence of a functional Min system bacteria will frequently divide close to one of the cell poles and generate the so-called minicells, small DNA-less cells that are incapable of further reproduction [12], [13]. The remarkable spatiotemporal fidelity of bacterial division, which is always symmetrical and in sync with DNA replication, can be explained by the joint action of the Min system and that of the nucleoid occlusion proteins, such as Noc in *B. subtilis* and SlmA in *E. coli*, which prevent FtsZ assembly over unreplicated nucleoids [14], [15].

The core of the Min system is comprised of the proteins MinC and MinD, which are widely distributed among bacteria. MinC is a cytoplasmic protein that has the ability to inhibit FtsZ polymerization in vitro and, thus, represents the ultimate effector of Min function [16], [17]. MinD, on the other hand, is a membrane associated ATPase of the ParA family that forms a complex with MinC and helps recruit it to the membrane [18]–[21]. The Min system exhibits site selectivity because the MinCD complex is itself localized to the cell poles. Localization occurs by alternative mechanisms and requires different partner proteins in different bacteria. In *B. subtilis*, localization of MinCD requires DivIVA, a pole-marking protein conserved in Gram-positive bacteria [22]–[26]. DivIVA associates with the bacterial cell poles probably because the protein has affinity for negatively curved membranes [27], [28]. Once at the cell pole, DivIVA functions as a landmark that guides the recruitment of several other proteins that need to be at the cell poles to exert their function. Polar recruitment of proteins by DivIVA can involve direct or indirect interactions. In the case of MinCD, the recruitment is indirect and requires MinJ, an integral membrane protein that serves as a bridge between MinCD and DivIVA [29], [30]. In contrast, in *E. coli*, polar localization depends on the protein MinE and is achieved dynamically, by the oscillation of the MinCD complex from one extreme of the cell to the other with a period of a minute [31]. This oscillation is an example of a spontaneous pattern formation process that involves the ATP-dependent interaction of MinD with itself and with the cytoplasmic membrane, and is set in motion by MinE, which interacts with MinD and stimulates its ATPase activity and favors its dissociation from the membrane [9], [32].

How MinC interacts with FtsZ and affects its polymerization has been studied in more detail in *E. coli*. Initial biochemical experiments revealed that MinC (in the form of a MalE-MinC fusion) inhibited the sedimentation of FtsZ polymers but did not inhibit FtsŹs GTPase activity [16]. This indicated that MinC does not inhibit the head-to- tail polymerization of FtsZ subunits into its basic (proto)filaments, but, instead, prevents the assembly of FtsZ into higher order structures, such as bundles of filaments. Indeed, EM and rheology measurements showed that FtsZ polymers formed in the presence of MinC are less bundled, and have fewer lateral contacts and interconnections [33]. In addition, FtsZ filaments became shorter and more curved, suggesting that MinC is also capable of destabilizing FtsZ filaments to some extent [33]. Similar results were obtained for MinC of *B. subtilis* [17]; thus, the overall mode of action of MinC is evolutionarily conserved. Concomitantly, in vivo experiments demonstrated that inhibition of Z ring formation requires the synergistic action of the two domains of MinC. MinC can be divided into N-terminal and C-terminal domains of similar sizes but different functions [34], [35]. The C-terminal domain (MinC_C_) is conserved among Gram negative and Gram positive bacteria and is the portion of the protein involved in the interaction with MinD [34]. The N-terminal domain (MinC_N_), on the other hand, is highly divergent, to the point that the Pfam entry describing it (MinC_N, accession PF05209) does not recognize MinC from Gram-positive bacteria. Mutations in either domain render MinC nonfunctional and lead to minicell formation [36]. Nevertheless, each domain by itself still retains the ability to inhibit Z ring formation when overexpressed [34], [37], [38]. Recent genetic experiments shed light on this behavior by showing that each MinC domain binds to a different portion of FtsZ [38], [39]. The structure of FtsZ strongly resembles that of tubulin, and is composed of two globular domains (the N and C terminal domains) followed by an unstructured tail containing a conserved 15 to 17 aminoacid peptide at its very C-terminus (the “C-Terminal Peptide” or CTP) [40], [41]. The CTP does not play a role in polymerization, but is essential for the division process notably as the binding site for FtsZ modulators such as FtsA, ZipA, EzrA and SepF [7]. MinC_N_ was shown to interact with the H10 helix at the globular C-terminal subdomain of FtsZ [39], whereas MinC_C_ recognizes a conserved 15 residue patch in the CTP tail of FtsZ [38]. Because helix H10 lies on the FtsZ-FtsZ polymerization interface, this could explain why binding of MinC, and, more specifically, MinC_N_, would promote weakening of FtsZ filaments and disrupt Z ring formation. The binding site of MinC_C_ can also be rationalized to explain the inhibitory effect of this domain. Because FtsŹs CTP is also the binding site for FtsA and ZipA, two key organizers of the Z ring in *E. coli* [42], competition with these modulators could explain why MinC_C_ inhibits Z ring formation when overexpressed. However, at physiological levels, the most likely role of the interaction between MinC_C_ and the CTP of FtsZ is to target MinC_N_ to FtsZ polymers. Thus, the mechanism of Z ring inhibition by MinC involves two simultaneous interactions of MinC with FtsZ, with MinC_N_ binding to a region of FtsZ that is important for polymer assembly and MinC_C_ contributing to the targeting of MinC to FtsZ.

Here we describe genetic and biochemical experiments that allowed the identification of the binding site(s) of MinC on *B. subtilis* FtsZ. These experiments suggest that the MinC binding site in *B. subtilis* FtsZ has the same bipartite layout as in *E. coli* FtsZ, involving residues in the vicinity of helix H10 and in the proteińs CTP. Significantly, however, the residues important for MinC binding in the H10 helix region of *B. subtilis* FtsZ were not the same as those in *E. coli* FtsZ. Whereas in *E. coli* these residues were located on the bottom surface of H10 and faced the FtsZ-FtsZ binding interface, in *B. subtilis* these residues were on the “side” of the molecule and next to the point where the unfolded CTP emerges from the FtsZ structure. This suggests that the mechanism of MinC inhibition of FtsZ polymerization differs in *B. subtilis* and *E. coli*.

## Methods

### General Methods

Strains and plasmids are described in Table S1 and their construction is detailed in the supplemental material. All strains were grown in LB medium at 37°C and supplemented with the following concentrations of antibiotics when necessary: 5 µg/mL for chloramphenicol, MLS (1 µg/mL of erythromycin plus 25 µg/mL of lincomycin); 100 µg/mL of spectinomycin; 10 µg/mL of kanamycin; and 10 µg/mL of tetracycline. Concentrations of inducers isopropyl-β-D-thiogalactopyranoside (IPTG) and xylose, when used are reported in figure legends.

### DNA Methods

Mutations in pAB20, pAB30 and pAB31 were constructed using site-directed mutagenesis (QuikChange; Stratagene) with Phusion DNA polymerase (NEB). Oligonucleotide primers were purchased from IDT (Coralville, IA) and are listed in Table S2. Sequencing was carried out using the Big Dye terminator cycle sequencing kit (Applied Biosystems) by the sequencing service of the Departamento de Bioquímica, IQ-USP.

### *ftsZ* Mutant Library

The *ftsZ* mutant library was constructed by Error-Prone PCR. The reaction conditions were as follows: 100 ng gDNA, 75 mM Tris-HCl (pH 8.8 at 25°C), 0.1% Tween 20, 20 mM (NH_4_)_2_SO_4_ (Taq Buffer Fermentas), 0.5 µM of each primer (OFG63 and OFG178), 7 mM MgCl_2_, 0.5 mM MnCl_2_, 1.5 mM dTTP, 1 mM dCTP, 0.2 mM dGTP, 0.6 mM dATP and 2U of Taq polymerase, in a final volume of 50 µl. Cycling parameters were: 1 cycle of 93°C for 3 min; 30 cycles of 93°C for 1 min, 55°C for 1 min, 72°C for 5 min; 1 cycle of 72°C for 10 min. The 1149 bp mutagenized DNA fragment containing the *B. subtilis ftsZ* gene without the ATG start codon was ligated into plasmid pDG1515 and transformed into *E. coli* giving rise to approximately 10^5^ colonies. Colonies were pooled and their plasmids were extracted to create a library.

### Fluorescence Microscopy

Microscopy was performed on a Nikon Eclipse Ti microscope, equipped with GFP BrightLine® and mCherry BrightLine® Filter Sets (Semrock), a Plan APO VC Nikon 100X objective (NA = 1,4), a 25 mm SmartShutter and a Andor EMCCD i-Xon camera. Exposure times varied from 0.3 to 1 s. Cells were grown to exponential phase and incubated in chambers with LB plus 1% agarose. Membrane stain FM5-95 (final concentration of 50 µg/mL; Molecular Probes) and 0.5 mM IPTG were added to the solidified LB, where indicated. All images were captured using NIS software version 3.07 (Nikon) and processed with ImageJ software (http://rsb.info.nih.gov/ij/).

### Purification of His_6_-FtsZ, His_6_-MinC and MinC-His_6_

The *ftsZ* gene and its mutants were cloned into the expression vector pET28a (Novagen) and transformed into BL21(DE3)-RIL cells and the His-FtsZ proteins were purified by two-step ammonium sulfate and GTP+Ca^2+^ precipitation [43]. The *minC* gene and its mutants were cloned into the expression vectors pET28a and pET24b (Novagen) and transformed into BL21(DE3)-RIL cells. Cell extracts were made in TKEG buffer and subjected to a 100.000× g spin and His-MinC and MinC-His were purified from the soluble fraction by Ni^++^-affinity chromatography. His-MinC and MinC-His were eluted from the column applying an imidazole gradient (100 mM to 1 M) in TKEG buffer. Imidazole was subsequently removed by desalting columns and the proteins were stored in TKEG. All proteins were quantified by the bicinchoninic acid (BCA) method, using bovine serum albumine (BSA) as the standard.

### GTPase Activity and Critical Concentration

GTPase activity was determined using the malachite green assay in 96-well plates. Proteins were tested from 0.5 µM to 10 µM in MMKE buffer (50 mM MES pH 6.5, 5 mM MgCl_2_, 50 mM KCl and 1 mM EDTA). The reaction was started by adding GTP (1 mM) at 37°C and stopped in intervals of 2.5 min by adding the color reagent (0.035% malachite green, 8.5% ammonium molybdate, 1 M HCl, filtered through a 0.45 µm filter). After 10 min, the A_650_ was measured using a Synergy™ HT plate reader (Bio-Tek). Using a KH_2_PO_4_ standard curve, a graph of phosphate release over time was plotted and the slope of the linear region was used to determine the hydrolysis rate. The hydrolysis rate at different protein concentrations was used to determine the critical concentration (Cc) of the different mutants.

### Light Scattering

Light scattered by FtsZ polymers was measured using in a Hitachi F-4500 fluorimeter. Excitation and emission wavelengths were set to 350 nm, with slit widths of 3.5 nm, and the photomultiplier tube at 950V with a scan rate of 60 nm/s. Protein was incubated in 150 µL of polymerization buffer (50 mM MES/NaOH pH 6.5, 133 mM KCl, 0.6 mg/mL DEAE and 10 mM MgCl_2_) at 30°C until baseline stabilization. After the baseline stabilized, GTP was added to 2 mM and the change in scattering was followed for the next 30 minutes. MinC was added to reactions either before or after the addition of GTP with similar results. Buffer effects were excluded by inclusion of MinC storage buffer to a similar volume as the maximum amount of MinC added.

### Transmission Electron Microscopy

For visualization in negative stain, FtsZ samples at a concentration of 5 µM were incubated in polymerization buffer (50 mM MES/NaOH pH 6.5, 100 mM KCl, and 10 mM MgCl_2_) for 5 min at 37°C after addition of 2 mM GTP. FtsZ-MinC samples were prepared in a molar ratio of 1∶3 and incubated for 3 min in the polymerization buffer, before adding GTP. Holey carbon coated electron microscope grids (with the holey carbon on its bottom face and a thin continuous carbon film on its top surface) were glow discharged. A 3 µl droplet of the sample was deposited onto a grid for 30 sec and blotted. A 3 µl of 2% uranyl acetate was added, blotted and air-dried. Images were recorded at −3 µm defocus and 60,000× magnification with a Jeol JEM-2100 operating at 200 kV and equipped with a TVIPS F-416 CMOS camera.

### Trp Fluorescence Measurements

Intrinsic fluorescence of tryptophan mutant MinC Y44W was monitored in the presence of wild-type and mutant FtsZ in Tris/HCl 20 mM, KCl 100 mM, EDTA 5 mM, pH 7,5. The tryptophan fluorescence was measured using a Hitachi F-4500 fluorescence spectrophotometer. The excitation wavelength was 295 nm, and the emission data were collected between 320 and 360 nm. All measurements were recorded in triplicate. The net MinC fluorescence intensity in the presence of FtsZ was determined using the following equation: (F_MZ_ – F_Z_) ^*^ V_total_, where F_MZ_ is the fluorescence of the MinC+FtsZ mixture, F_Z_ is the fluorescence of the same amount of FtsZ in the absence of MinC, and V is the volume of the reaction. Thus, all MinC fluorescence spectra were corrected for background fluorescence from FtsZ and buffer components, and for volume changes.

## Results

### A Genetic Screen for Min Resistant FtsZ Mutants

To determine the binding site for MinC on *B. subtilis* FtsZ we carried out a genetic screen to identify FtsZ mutants that made cells resistant to MinD overexpression, a condition that causes filamentation and lethality in *B. subtilis* [43]. Because lethality produced by MinD overexpression is strictly dependent on, and promoted by, MinC [43] we reasoned that resistance to MinD overexpression should select for mutations that disrupt the interaction between FtsZ and MinC. A library of approximately sixty thousand independent FtsZ mutants generated by error prone PCR (see methods for details) was introduced into a strain containing an IPTG inducible copy of MinD (FG249) and selected in the presence of 250 µM IPTG, a condition that ordinarily kills all cells bearing wild-type FtsZ. We recovered 51 colonies out of 6×10^4^ transformants, a frequency of Min resistance of about 0.1%. We performed backcross experiments to confirm that resistance was linked to FtsZ and sequenced the *ftsZ* allele in all of those mutants that were linked. Sequencing revealed a total of 13 single substitutions (Table 1). Because several of these were isolated multiple times, we believe our screen reached near saturation. We have also included in our dataset one mutant (T111A) that was originally isolated in a screen against a different FtsZ modulator (ZapA - Alexandre Bisson-Filho, Frederico Gueiros-Filho, unpublished results) but that was found to also confer resistance to Min.

**Table 1:** Resistance of FtsZ mutants.

	MinCD	MciZ	ZapA-MTS
WT	−	−	−
L69S	+	+	+
T111R	+	−	−
T111A	+	+	+
T232I	+	−	+
K243R	+	−	−
I245F	+	−	−
D255V	+	−	−
V260A	+	−	−
V282A	+	−	−
A285T	+	−	−
D287V	+	−	−
I293T	+	+	−
V310A	+	−	−
R376T	+	−	−

There are reports in the literature that FtsZ mutations that promote resistance to its inhibitors do not always disrupt the binding between FtsZ and the inhibitor. These mutations usually affect the GTPase activity of FtsZ and are “promiscuous”, because they will also confer resistance to other unrelated inhibitors. One example are certain mutations in *E. coli* FtsZ that were selected for resistance to SulA but that also make the cell resistant to MinC [44], [45]. Because of this precedent, we subjected our mutants to a first filter to sort them between those that were promiscuous and those that were specific to Min. This was done by testing if our mutations conferred cross-resistance to two unrelated FtsZ inhibitors, the peptide MciZ [46], and an altered version of ZapA (ZapA-MTS). ZapA is normally a positive modulator of FtsZ. However, we found that appending a membrane targeting sequence to the C-terminus of ZapA turned the protein into a potent inhibitor of cell division (Alexandre Bisson-Filho and Frederico Gueiros-Filho, unpublished results). FtsZ mutations were transferred to strains capable of overexpressing either MciZ (AB52) or ZapA-MTS (AB53) and resistance was tested in plating experiments that are summarized in Table 1 (see also Figure S1 for the original data). Nine out of the thirteen mutants analyzed were specific to Min. Among the non-specific mutants, one (L69S) was resistant to all three inhibitors, whereas the other two showed cross resistance to either MciZ (I293T) or ZapA-MTS (T232I). One curious case was residue T111: substitution of this threonine with arginine (T111R) resulted in a protein that seemed specifically resistant to Min, whereas substitution to alanine (T111A) led to a protein that was cross-resistant to all three modulators.

**Figure 1:**
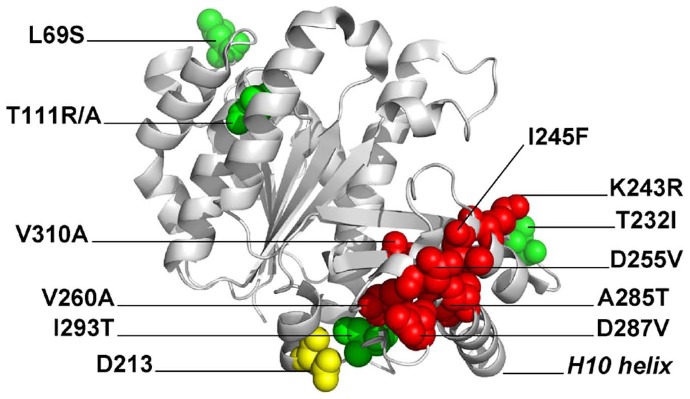
Mapping of the mutations conferring MinC resistance onto the structure of FtsZ. The structure of *B. subtilis* FtsZ was obtained from the Protein Data Bank (2VAM) [75]. Residues marked in red represent those altered in MinC-specific mutants, whereas residues in green are altered in promiscuous mutants. Polymerization occurs by interaction between the top and bottom surfaces of the molecule, following an imaginary vertical axis defined by catalytic residue D213, marked in yellow.

Mapping the mutations onto the FtsZ crystal structure revealed that most substitutions were located in the globular C-terminal subdomain of FtsZ (Figure 1), where they clustered around the H9 and H10 helices, the same region that has been identified as one of the two regions of FtsZ bound by *E. coli* MinC [39] (see an alignment in Figure S2 for the location of the mutations relative to the secondary structure of *B. subtilis* FtsZ). In addition, three mutations (L69S, T111A and T111R) mapped to the N-terminal subdomain and one (R376T) mapped to FtsŹs CTP and, thus, does not appear in the crystal structure. Interestingly, there was a striking correlation between the type of mutation (specific versus promiscuous) and where they localized. With the exception of T232I, all other promiscuous mutations localized to the surfaces of FtsZ involved in GTP binding and formation of the FtsZ-FtsZ longitudinal bond (here we treated the T111 position as promiscuous because there is at least one substitution in this position that produces resistance to multiple modulators). This suggests that these mutations affect the GTPase activity and/or affinity of FtsZ for itself. Indeed, substitution of *E. coli* ´s residue L68 (equivalent to *B. subtilis* L69– see also the alignment in Figure S2) to tryptophan causes an increase in the affinity between FtsZ monomers and a 10 fold decrease in the critical concentration for polymerization [47], [48]. In contrast, the Min specific mutations were found exclusively in the neighborhood of helices H9 and H10. In addition, the isolation of a mutation in the CTD of *B. subtilis* FtsZ suggests that, like in *E. coli*, the interaction between FtsZ and MinC may involve two contact sites between the proteins. We chose to further characterize two of the most surface exposed of the Min-specific mutations (K243R, D287V) in the vicinity of the H10 helix, as well as the R376T mutation in the CTP because they seemed like the best candidates for being part of the MinC binding site(s). For the sake of comparison, we also characterized a mutation that was promiscuous and located in the N-terminal subdomain of FtsZ, T111A, and thus unlikely to be part of the MinC binding site.

### Microscopic Characterization of Min Resistant Mutants

We examined mutants T111A, K243R, D287V and R376T by fluorescence microscopy to assess how these substitutions affected division both in the absence and in the presence of MinD overexpression.

**Figure 2:**
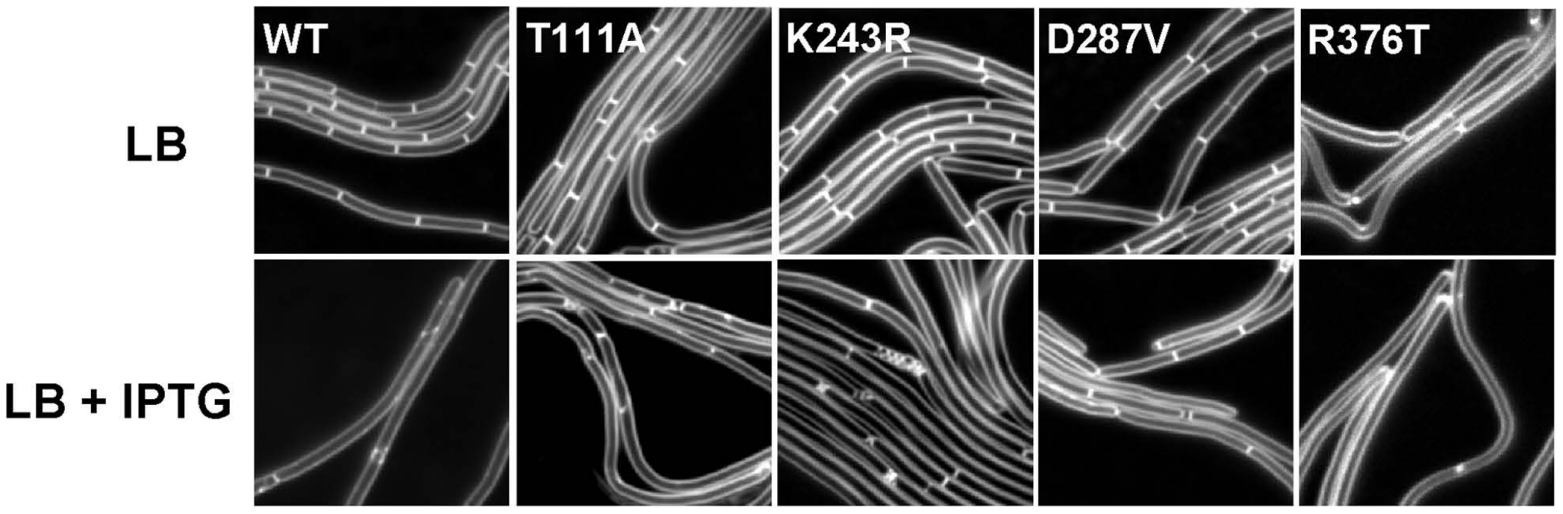
Cell division phenotype of FtsZ mutants. Strains containing an IPTG inducible allele of *minD* (*thrC*::P_spac-hy_-*minD*, *erm*) plus either wild-type or Min-resistant *ftsZ* alleles were grown on LB-agar chambers with or without 1 mM IPTG for 2 hours and imaged by microscopy. Membranes were stained with FM 5–95. Arrowheads point to a typical minicell (yellow) and an abnormally sized minicell (red). Strain numbers: FtsZ wild-type (AB164), T111A (AB70), K243R (AB168), D287V (AB174), R376T (AB177). The scale bar corresponds to 5 µm.

In the absence of MinD overexpression (Fig. 2, Table 2, -IPTG column), most mutants had an average cell size that was close to that of the wild-type, suggesting that these alterations in FtsZ did not significantly disrupt the proteińs normal function. The exception was R376T, which was clearly impaired for division, with cells that were about 2 fold longer than the wild type (9.6 versus 4.4 µm) and prone to lysis as judged by their non-homogenous membrane staining. R376T has a substitution in one of the conserved residues of the CTP that are important for binding of proteins such as FtsA, SepF and EzrA (and ZipA in *E. coli*) [49]–[52] that promote Z ring formation in vivo and this may explain its impaired division.

**Table 2:** FtsZ mutant measurements.

	Average Size (µm)		% Minicells	
	− IPTG	+ IPTG	− IPTG	+ IPTG
WT	4.4	17.1	0	0
ΔminCD	8.1	–	16.5	–
T111A	5.9	9.8	16.0	14.0
K243R	5.1	7.9	2.5	17.0
D287V	4.7	6.2	3.5	5.0
R376T	9.6	13.6	8.0	8.5

Induction of MinD overexpression for two hours (Fig. 2, Table 2, +IPTG column) led to a five fold increase in the length of wild-type cells. As expected, the same treatment had a much less severe effect in the Min resistant mutants. Importantly, however, most Min resistant mutants were not completely insensitive to MinD overexpression, since their average length increased by 32% to 66%.

Another piece of information extracted from the microscopy data was how similar the FtsZ mutants were to a Min null mutant (in this case a MinCD deletion). FtsZ mutants that become completely insensitive to Min should phenocopy the MinCD deletion mutant. Focusing on the frequency of minicelling, T111A is the mutant that is most similar to the MinCD deletion. Closer inspection of the images, however, revealed that most minicells in the T111A mutants are significantly larger than the typical minicells produced in a Min mutant (see arrowheads in Fig. 2). This suggests that they derive from altered properties of FtsZ that go beyond the interaction with Min, as also suggested by the fact that this mutant is cross-resistant to several other FtsZ modulators. Among the remaining mutants, K243R and D287V showed a frequency of minicelling in the range of 3%. Importantly, the minicells in these mutants had the size and appearance of typical minicells. The 3% frequency of minicelling is lower than the 16.5% observed for the MinCD mutant, nevertheless, it is significantly higher than the wild type situation. This is in line with the idea that K243R and D287V are partially insensitive to MinCD. A similar situation was reported for *E. coli*, where mutants selected for resistance to MinC were also only partially resistant [39]. We have also noticed that induction of MinD led to an increase in minicell frequency in the case of the K243R mutant, and perhaps in the case of the D287V mutant as well. This may indicate that division becomes favored in the polar sites when MinCD is present all over the cell, a situation similar to a *divIVA* mutant [53], [54], and reinforces the idea that the K243R mutant, in particular, is still somewhat sensitive to MinC.

### FtsZ Mutants are Insensitive to MinC *in vitro*

To investigate their biochemical properties, the T111A, K243R, D287V and R376T proteins were overexpressed in *E. coli* and purified. We also included in our in vitro experiments a truncated version of FtsZ, lacking the entire CTP (FtsZΔCTP). This mutant, which does not support division in vivo and could not have been recovered in our screen, was chosen to corroborate the role of the CTP in the FtsZ-MinC interaction. Even though the proteins had N-terminal histidine tags we opted to purify them by GTP induced polymerization followed by sedimentation (see Methods) because this method selects for active protein and in our hands produced FtsZ preparations that polymerized much more robustly than when the same protein was purified by metal affinity chromatography. Comparison between untagged and his-tagged versions of wild-type FtsZ showed no noticeable difference in polymerization behavior and MinC sensitivity (data not shown). We have also overexpressed and purified wild-type MinC and the mutant MinC19 (MinC G13D), the *B. subtilis* equivalent of the well characterized nonfunctional MinC19 mutant of *E. coli* [16]. Both MinC proteins have C-terminal histidine tags and were purified by metal affinity chromatography. All purified proteins were correctly folded as assessed by CD spectroscopy (Figure S3). The minor difference in the CD spectrum of the FtsZΔCTP mutant can be explained because this protein is missing a fragment of its C-terminus.

We used light scattering to measure FtsZ polymerization *in vitro* in a buffer containing 50 mM MES/NaOH pH 6.5, 133 mM KCl, 0.6 mg/mL DEAE-dextran and 10 mM MgCl_2_. We chose this condition because it favors bundle formation by FtsZ, a situation that has been shown to facilitate visualization of MinC inhibition of FtsZ assembly [17]. Initial experiments showed that all mutant FtsZ proteins were capable of polymerizing when assayed at 5–7 µM (Figure S4). This was expected because most of the mutants exhibited robust division *in vivo* (Figure 2, Table 2). Interestingly, even the R376T protein polymerized well in vitro, supporting the idea that the division defect of this mutant is due to problems interacting with FtsZ modulators. Nevertheless, the light scattering signal tended to be lower for the mutant proteins when compared with the wild-type (Figure S4). Because the light scattering signal is sensitive to both mass and size of the polymers being monitored, we also evaluated the polymerization of all mutants by sedimentation. This showed that the total mass of polymerized FtsZ was essentially the same for mutant and wild-type FtsZ (Figure S5). This result, and the measurements of critical concentrations of polymerization shown below (Table 3), indicate that the mutant FtsZ are generally as prone to polymerization as the wild-type protein. We cannot rule out, however, that the mutations conferring resistance to MinC have affected to some extent the architecture of the polymers formed.

**Table 3:** GTPase activity and Cc of FtsZ mutants.

	GTPase FtsZ^−1^ min^−1^	Cc (µM)
WT	2.6±0.2 (100±8%)	1.6
T111A	0.3±0.01 (13±3%)	3.3
K243R	2.1±0.1 (81±5%)	2.1
D287V	4.3±0.2 (165±5%)	1.9
R376T	2.4±0.1 (89±4%)	1.5
ΔCTP	2.3±0.1 (88±4%)	0.8

**Figure 3:**
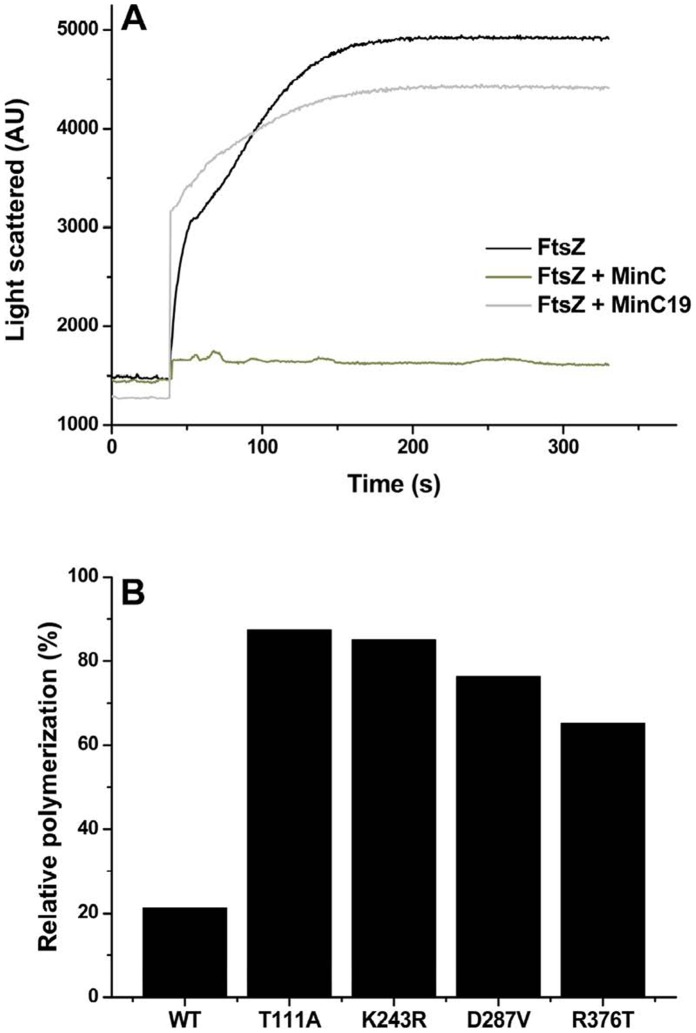
Polymerization of FtsZ mutants in vitro is not affected by MinC. A. Representative light scattering trace of an experiment performed with wild-type FtsZ in the absence of inhibitor and in the presence of MinC or MinC19. Reactions contained 7 µM of FtsZ and 20 µM of MinC or MinC19 in Mes/NaOH 50 mM, MgCl_2_ 10 mM, KCl 133 mM, DEAE-dextran 0.6 mg/mL, pH 6.5. B. Experiments similar to A were performed and the inhibition of the polymerization of each FtsZ mutant by MinC was quantified relative to the maximum light scattering signal in the absence of MinC. The original light scattering traces corresponding to this graph can be found in Fig. S4. Measurements were repeated at least three times for each FtsZ mutant with similar results.

Next, we used the light scattering assay to investigate the effect of MinC on FtsZ polymerization. Polymerization of wild-type FtsZ was inhibited by MinC in a dose dependent manner, with a 3 fold molar excess of MinC (21 µM) resulting in 80–90% inhibition (Figure 3A). This is similar to previous data that showed that MinC has maximal activity when in molar excess [16], [17]. Importantly, inhibition by MinC in our assay was specific because the same amount of the MinC19 mutant protein did not inhibit FtsZ polymerization (Figure 3A). In contrast to the effect of MinC on wild-type FtsZ, adding MinC to polymerization reactions containing the different FtsZ mutants had little effect. Most mutants polymerized to 80–90% of the levels achieved in the absence of MinC. R376T showed the highest residual inhibition, achieving only 70% of the light scattering signal in the absence of MinC (Figure 3B and S4).

**Figure 4:**
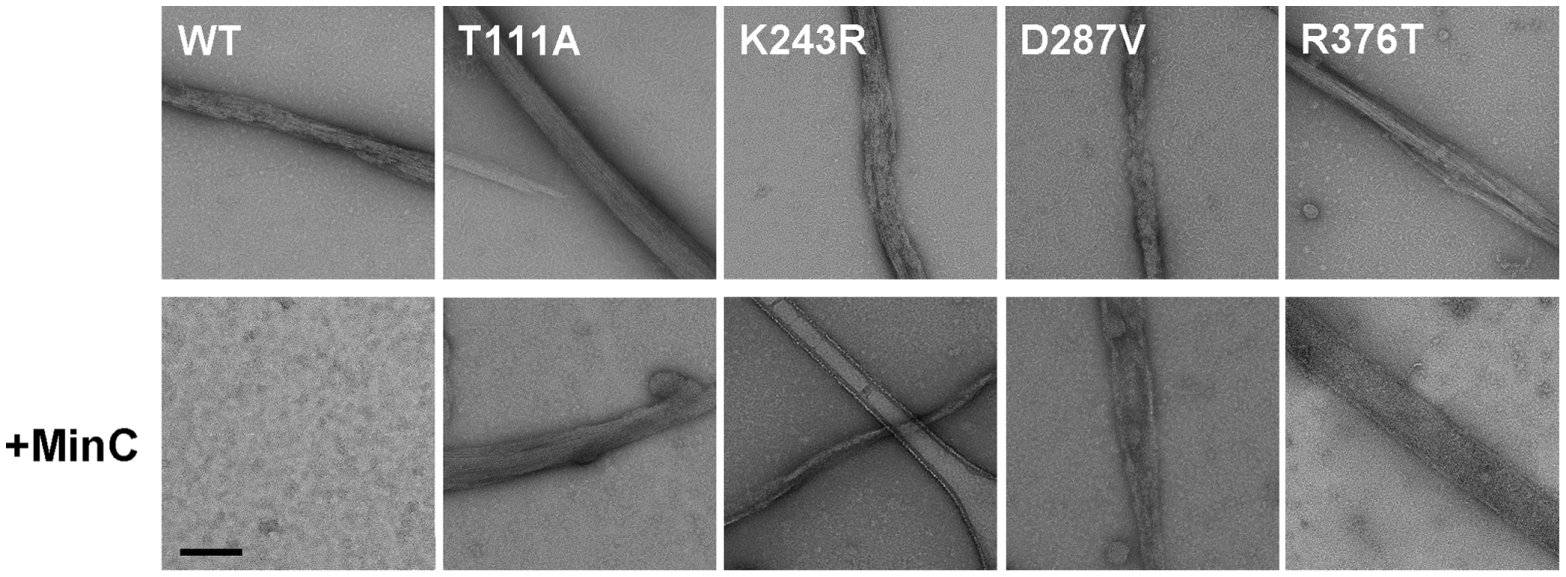
Electron microscopy of FtsZ polymers formed in the presence or absence of MinC. Purified wild-type and mutant FtsZ were polymerized in reactions containing 5 µM of FtsZ, Mes/NaOH 50 mM, MgCl_2_ 10 mM, KCl 133 mM, pH 6.5, and either no (top row) or 20 µM MinC (bottom row). Polymerization reactions were spotted on carbon coated 400 mesh copper grids and stained with uranyl acetate for transmission electron microscopy. Scale bars equal 200 nm.

We have also used EM to investigate the effect of MinC on the polymerization of mutant FtsZ. EM was carried out in the absence of DEAE-dextran, a more physiological condition, and showed that wild type FtsZ polymerized into a mixture of individual protofilaments and large protofilament bundles (Figure 4). Similarly to the wild-type protein, mutant FtsZ also formed individual protofilaments and large protofilament bundles upon polymerization. Addition of MinC to the reaction abolished bundle formation by the wild-type protein (Figure 4, +MinC row). In contrast, bundle formation by mutant FtsZ was basically insensitive to MinC (Figure 4, +MinC row), although the bundles formed in the presence of MinC were somewhat less regular than those in control reactions, perhaps because of residual MinC binding to the mutant FtsZ. Thus, based on two independent *in vitro* assays, we conclude that every one of our FtsZ mutants is resistant to the biochemical activity of MinC.

### Determination of the GTPase Activity and Critical Concentration of FtsZ Mutant

Resistance to MinC could arise because a mutation disrupted contact between FtsZ and MinC, or indirectly, if the mutation alters the polymerization properties of FtsZ. Indirect resistance is often associated with a significant decrease in the GTPase activity of FtsZ [44], [45]. Thus, to try to distinguish the biochemical mechanism behind resistance in our mutants we measured their GTPase activity using a malachite green assay. This showed that the T111A mutant had tenfold lower GTPase activity (Table 3, Figure S6), consistent with other data suggesting that it is an indirect mutant. All other mutants had essentially normal GTPase activity, varying from 80 to 140% of the activity displayed by the wild-type protein (Table 3, Figure S6).

We also measured the critical concentration of polymerization of the FtsZ mutants by assaying their GTPase activity over a range of FtsZ concentrations. These experiments showed that the critical concentration of mutant proteins was not significantly altered (Table 3, Figure S7), ranging from two fold lower than the wild-type, in the case of FtsZΔCTP, to two fold higher than the wild-type, in the case of T111A. Most of the mutants, however, had critical concentrations very similar to the wild-type, implying that alterations in the affinity of the FtsZ-FtsZ interaction cannot explain their resistance to MinC.

### Mutants on the H10 Helix and CTP of FtsZ Exhibit Reduced Binding to MinC

We have exhaustively attempted to measure binding between FtsZ and MinC by co-sedimentation, pull-down and far-Western assays but failed (data not shown). These assays were successful in demonstrating a direct interaction between FtsZ and MinC in the case of the *E. coli* proteins [16], [33], [39] but previous *in vitro* work with *B. subtilis* FtsZ and MinC also failed to reproduce the *E. coli* results [55]. The discrepancy between the behavior of the *E. coli* and *B. subtilis* proteins may reflect real differences in their biochemical properties, or may be related to the different tags used to purify MinC. The MalE tag used with *E. coli* MinC has been shown to exhibit non-specific binding to *B. subtilis* FtsZ [17] and, thus, it is conceivable that this tag may stabilize the FtsZ-MinC interaction in the *E. coli* studies.

**Figure 5:**
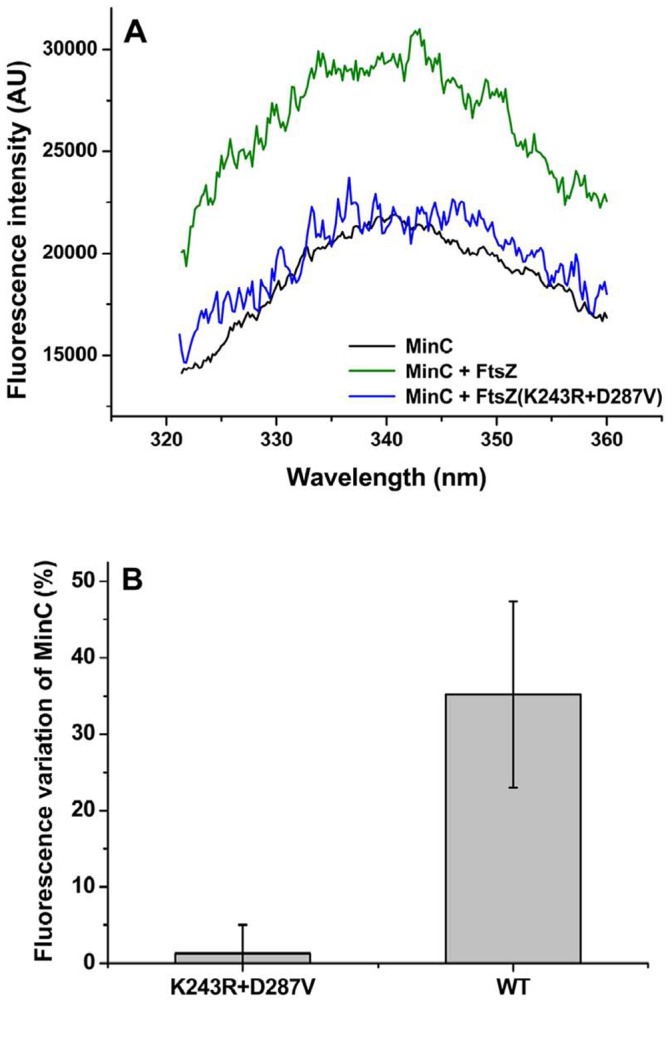
Trp fluorescence measurements of MinC binding to mutant FtsZ. Wild-type or double-mutant (K243R,D287V) FtsZ **(**2 µM) were mixed with 1 µM MinC Y44W in buffer Tris/HCl 20 mM, KCl 100 mM, EDTA 5 mM, pH 7,5 and fluorescence emission at 320–360 nm was recorded. A. Representative trace of one experiment. B. Averaged quantitation of 3 experiments, with standard deviations.

We have succeeded in using intrinsic tryptophan fluorescence to monitor the interaction between *B. subtilis* FtsZ and MinC. Trp fluorescence is a widely accepted way to demonstrate protein-protein interactions and has the advantage of being a homogenous solution assay. Because *B. subtilis* MinC lacks tryptophan we employed site-directed mutagenesis to introduce tryptophan residues in two positions in the N-terminal domain of MinC (Y44 and F62). These positions were chosen because they are surface exposed on a model of *B. subtilis* MinC structure, and originally contained aromatic amino acids suggesting that a substitution to tryptophan would be tolerated. Testing of these Trp mutants revealed that only Y44W remained functional, as determined by the inhibition of FtsZ polymerization in light scattering assays (Figure S8). Mixing of MinC Y44W with FtsZ led to a 50% increase in Trp emission intensity. This suggests that the tryptophan at position 44 is becoming less solvent exposed in the presence of FtsZ and could indicate that this residue participates in the contact between MinC and FtsZ. The increase in tryptophan fluorescence of MinC Y44W was saturable (Figure S9), as expected if it were reporting the binding interaction between MinC and FtsZ. The low signal to noise ratio of our assay prevented us from detemining a precise K_d_ from titrations like the one in Figure S9, but the tendency to saturation over 1 µM FtsZ implies that the K_d_ for the interaction between FtsZ and MinC in *B. subtilis* will be around 1 µM. This is not very different from the value (6 µM) measured by Shen and Lutkenhaus [39] for *E. coli* using SPR. Next, we used the Trp fluorescence of MinC Y44W as a readout to measure binding between MinC and the FtsZ mutants. Experiments with single mutants showed a tendency towards loss of binding (data not shown) but the results were too variable to be conclusive, probably because our assay lacks sensitivity to detect moderate changes in binding affinity. To circumvent this problem, we constructed a double mutant, FtsZ K243R,D287V, combining two of the substitutions postulated to affect MinC-FtsZ contacts. Assaying this mutant at three protein concentrations (1, 2, and 3 µM) that approach or are above the saturating concentration for wild-type FtsZ showed essentially no change in MinC Y44W fluorescence (see Figure 5 for a representative experiment and Figure S10 for the average of all concentrations tested). This is consistent with the hypothesis that the Min-specific mutations we identified in our screen do indeed alter binding site residues in FtsZ.

## Discussion

We have used combined genetic and biochemical approaches to identify the binding site for MinC in *B. subtilis* FtsZ. By screening a comprehensive library of FtsZ mutants for substitutions that conferred resistance to MinC we identified a series of residues in the vicinity of helices H9 and H10 in the C-terminal subdomain of FtsZ. We also found that a substitution on the CTP of *B. subtilis* FtsZ was capable of generating MinC resistance. Previous work from the Errington lab [56] had already identified three of the same mutations found in our screen (*ftsZ4*– A285T; *ftsZ3*– V260A; *ftsZ24*– I245F) as likely insensitive to MinC based on the observation that they caused cells to resemble a Min mutant and produce minicells. More recently, the Scheffers group confirmed this suspicion by reisolating some of the mutations described in the Errington paper in a screen for MinCD resistance [55]. However, biochemical characterization of these proteins failed to provide a conclusive molecular explanation for their MinCD resistance [55]. Here we have confirmed and extended this work, by providing a thorough biochemical characterization of a group of Min resistant mutants that strongly implicate the H9–H10 helix region as part of the binding site for MinC in *B. subtilis* FtsZ. In addition, we have provided evidence for the first time that the CTP of FtsZ is involved in the interaction with MinC in *B. subtilis*.

Our data is consistent with a model in which the residues in the vicinity of helices H9–H10 and in the CTP of *B. subtilis* FtsZ are the contact sites for MinC. This conclusion is supported by the following evidence: First, mutations in these regions are associated with specific resistance to MinC and do not show cross-resistance to other FtsZ inhibitors (Table 1). Secondly, the GTPase activity and polymerization properties of these mutants are very similar to the wild-type (Figures 3, 4, S5, Table 3). This implies that their resistance is due to the loss of a MinC-FtsZ protein-protein contact, instead of being an indirect consequence of altered dynamics of FtsZ polymerization. Thirdly, mutants with alterations in these regions showed reduced interaction with MinC *in vitro*, as inferred from tryptophan fluorescence experiments (Figure 5).

**Figure 6:**
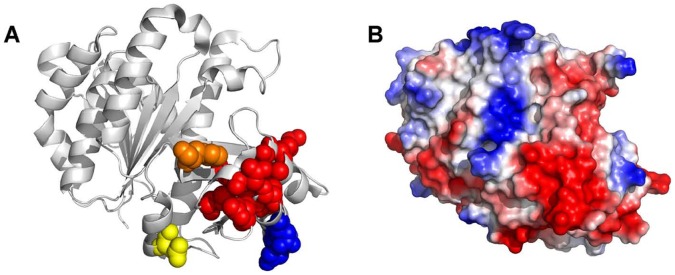
The binding site for MinC differs in *B. subtilis* and *E. coli* FtsZ. A. Comparison between mutations that promote MinC resistance in *B. subtilis* (red residues) and *E. coli* (blue residues) mapped onto the 2VAM FtsZ structure [75]. *E. coli* residue N280 corresponds to *B. subtilis* D280; E276 corresponds to Q276; R271 corresponds to S271. Two other important landmarks are highlighted in the structure: catalytic residue D213, shown in yellow, defines the center of the FtsZ-FtsZ interface (the polymerization axis follows a vertical line through this residue); and the point where the CTP should emerge from the structure (residue F315) is shown in orange. B. Surface electrostatic potential of *B. subtilis* FtsZ (2VAM) obtained with the “protein contact potential” tool of PyMOL. Note that the MinC binding site corresponds to a highly negative region of the FtsZ molecule.

Most of the mutations conferring MinC resistance identified in this study are located in surface residues that cluster at the C-terminal ends of helices H9 and H10 and the intervening loops between them. We also found two buried residues (V260 and V310) in this region that conferred MinC resistance. These residues are part of the beta sheet that lies underneath helices H9 and H10, and may affect the putative binding site for MinC by perturbing the overall topology of these helices. Importantly, the patch of mutated residues in helices H9 and H10 corresponds to a highly negative region of the FtsZ surface (Figure 6B), suggesting that the interaction between FtsZ and MinC has an important electrostatic component. Consistent with this, Scheffers [17] demonstrated that the *B. subtilis* FtsZ-MinC interaction is pH sensitive, being weaker at lower pH, and we found that the FtsZ-MinC interaction detected in our fluorescence experiments was inhibited by salt (Figure S11). Another interesting observation about the binding site for MinC in *B. subtilis* FtsZ is that the patch of negative residues on helices H9–H10 is located next to the region where the unstructured CTP emerges from the FtsZ molecule (residue F315, marked in orange in Figure 6A). Thus, the two regions of FtsZ recognized by MinC are close in space and may in fact need to come together to generate the MinC binding surface in FtsZ.

Our screen also identified a mutation in the CTP of *B. subtilis* FtsZ (R376T) as sufficient to confer resistance to MinC. Analogous mutations in *E. coli* also produce resistance to moderate overexpression of MinCD, even though they are still sensitive to overexpression of the N-terminal domain of MinC [38], [39]. The *in vitro* resistance of R376T to MinC (Fig. 3) mirrors its *in vivo* resistance, but the fact that this protein is the least resistant mutant *in vitro* may reflect that it should still retain contacts with the N-terminal domain of MinC. Residue R376T is part of the conserved core of the CTP motif of FtsZ. This arginine has recently been shown by X-ray crystallography to be part of the contacts between the CTP and FtsA in *Thermotoga maritima* [57] and to be critical for the interaction with SepF in *Bacillus subtilis* [58]. Thus, this may explain why mutation of this residue causes a significant impairment in *B. subtilis* division *in vivo*, even though the mutant protein polymerizes normally *in vitro* (see Tables 2 and 3). In *E. coli*, four CTP residues were found to confer MinC resistance (D373, I374, L378, K380) but R379 (the equivalent of R376 in *B. subtilis*) was not one of them. The lack of overlap between the CTP mutations is likely due to the superficiality of our sampling. Our screen was done against full length MinC, a situation that probably favors retrieval of mutations in the H9–H10 region since these do not seem to perturb the normal function of FtsZ, in contrast with the mutations in the CTP.

We also identified mutations that promoted MinC resistance because they altered the GTPase activity and, consequently, the dynamics of FtsZ polymerization. The two mutations that had demonstrated reductions in GTPase activity were T111A and L69S (Table 3 and data not shown). Mutations in residue L68 of *E. coli* FtsZ (equivalent to L69 in *B. subtilis*) had been shown before to lower GTPase activity 2 to 30 fold, depending on the substitution [59]. In contrast, the T111A mutation had never been reported before. Residue T111 is the second threonine in the conserved “tubulin signature motif” of FtsZ. It is located at the end of the T4 loop and may engage in interactions with the beta phosphate of GTP (in fact, the side chain hydroxyl of T111 seems to make a hydrogen bond to a water molecule that, in turn, makes an H bond to the beta phosphate in structure 2RHL of *B. subtilis* FtsZ [60]). The substitution of T111 for alanine lowers GTPase activity 10 fold and does not affect the critical concentration of the protein, as expected from the fact that this residue does not participate in the FtsZ longitudinal bond. Mutations in other canonical tubulin signature motif residues, such as G105S (the *Z84 E. coli* allele) and T108A (the *Z3 E. coli* allele), have been shown to also cause marked reduction in GTPase activity [61]–[63]. Among the mutations whose GTPase was not tested, I293T is located on the bottom surface of FtsZ, very close to loop T7, which contains critical residues for GTP hydrolysis. Mutations in residues next to I293 (W319Y of *Methanococcus jannaschii*, I294W of *E. coli*) showed 10 to 100 fold reduced GTPase [64], [65] and it is likely that I293T will have the same effect. Lastly, T232I is on a lateral surface of FtsZ, away from residues involved in GTP binding and hydrolysis. The only evidence suggesting it may affect GTPase activity is cross-resistance to another FtsZ modulator (ZapA-MTS). Better characterization of this mutant will be necessary before we can explain why it becomes resistant to MinC.

GTPase down mutations have been known for some time to cause resistance to FtsZ inhibitors [44], [66]. The requirement for a dynamic polymer is easier to rationalize for inhibitors such as SulA that bind to the polymerization interface of FtsZ [67]. In this case, a more stable FtsZ-FtsZ interaction should outcompete binding of the inhibitor. However, we postulate that a more stable FtsZ-FtsZ interaction may also lead to resistance to inhibitors that affect the higher order assembly of FtsZ polymers, such as MinC, by increasing the likelihood that FtsZ filaments will assemble into bundles or other multifilament structures. Bundling of filaments depends on weak lateral interactions between individual FtsZ subunits in the filaments. Because slowing down GTP hydrolysis leads to longer FtsZ filaments [68], this should facilitate bundle formation. In line with this idea, low GTPase mutants of FtsZ tend to assemble in vitro into larger and more regular structures than the wild-type protein (see for example the sheets produced by *Z84* [63]).

The identification of the MinC binding site in *B. subtilis* FtsZ should contribute to further the understanding of the mechanism of this inhibitor. In *E. coli*, MinC binds simultaneously to the CTP and the H10 helix of FtsZ. The interaction between the C-terminal domain of MinC and the CTP was proposed to target MinC to the Z ring, whereas the interaction of the N-terminal domain of MinC with the H10 helix of FtsZ would exert the inhibitory effect of MinC on FtsZ assembly. Because the residue conferring resistance to MinĆs N-terminal domain is part of the FtsZ-FtsZ interface and seems to contribute to polymer stability, inhibition by MinC in *E. coli* has been proposed to involve weakening of the bonds that form the protofilament [39]. Our findings establish that the overall architecture of the MinC-FtsZ interaction is similar in *B. subtilis*, involving the same two regions of FtsZ identified in *E. coli*. However, closer inspection of the H9–H10 mutations that confer MinC resistance in each species suggests that there are significant differences in the details of how MinC interacts with FtsZ. Whereas H10 mutations in *E. coli* affect residues involved in FtsZ-FtsZ contacts, in *B. subtilis* the residues affected will be surface exposed when the protein is polymerized (see Figure 6A for this comparison). Even though our screen was close to saturation, we cannot rule out that it missed mutations in the FtsZ-FtsZ interface similar to the ones described by Shen and Lutkenhaus [39]. Nevertheless, we find it more likely that our data reflects a real difference in the mechanism of MinC in *B. subtilis* and *E. coli.* There are several important differences in the biology of the Min system in *B. subtilis* and *E. coli*, including the topological specificity factors and the mechanism of polar localization of the Min proteins. In addition, recent work by the Levin lab demonstrated that there is significant variation in the polymerization properties of FtsZ of *B. subtilis* and *E. coli*, with the protein from *B. subtilis* being naturally more prone to bundling [69]. Furthermore, the N-terminal domain of MinC, which is the portion of the protein that binds to the H9–H10 region of FtsZ, is quite dissimilar between Gram-negative and Gram-positive bacteria (17% identity over 107 aminoacids), suggesting that the proteins evolved in response to different functional constraints, such as the ones just mentioned. Thus, we propose that Z ring inhibition in *B. subtilis* is a consequence of MinC preventing FtsZ filament bundling and does not involve the perturbation of longitudinal FtsZ-FtsZ contacts.

The proposed anti-bundling mechanism for *B. subtilis* MinC makes sense in light of the current models of Z ring formation. A key step in Z ring maturation is the compaction of FtsZ helices into a tight ring [70], [71], which is facilitated by proteins such as ZapA that bundle FtsZ filaments [72], [73]. Given the known antagonistic activities of ZapA and MinC detected in genetic and biochemical experiments [17], [33], [43], it seems reasonable to assume that preventing bundling should be sufficient to inhibit Z ring formation. Further support for the idea that bundling is crucial for Z ring formation comes from the observation that mutations in the lateral surfaces of FtsZ often generated proteins incapable of supporting Z ring formation *in vivo* [63], [74], and, more recently, that removing the positive residues on the CTP that facilitate bundling of *B. subtilis* FtsZ is sufficient to impair Z ring formation and division [69]. Even the proposed filament-breaking activity of *E. coli* MinC may in fact perturb Z ring formation by reducing the likelihood that the smaller filaments will form bundles. In this regard, it is noteworthy that *E. coli* seems to be more sensitive than *B. subtilis* to the absence of the bundling activity provided by ZapA [73].

In summary, we have identified the binding sites for MinC in *B. subtilis* FtsZ and found that they differ significantly from that in *E. coli*. This led us to propose that the mechanism of MinC action differs in both species, being primarily at the level of inhibiting FtsZ filament bundle formation in *B. subtilis*. In addition to advancing our understanding of the MinC protein of *B. subtilis*, our work highlights the variation that may exist in the biochemistry underlying the function of even conserved components of the divisome.

## Supporting Information

Figure S1 **Cross-resistance of FtsZ mutants.** Mutants were evaluated in three different strain backgrounds, each capable of overexpressing a different FtsZ modulator (top: MinD; middle: ZapA-MTS; bottom: MciZ). Strains were streaked onto a control plate or a plate containing inducer to promote overexpression of MinD, ZapA-MTS or MciZ. Growth was scored after overnight incubation at 37°C.
(TIF)

Figure S2 **Amino acid sequence alignment of FtsZ proteins, from** ***B. subtilis*** **(P17865),** ***E. coli*** **(P0A9A6) and** ***M. jannaschii*** **(Q57816) using ClustalW2.** Secondary structure prediction using Sequence Annotated by Structure-SAS (7) for *B. subtilis* FtsZ is shown above the alignment. The residues affected in the Min-resistant mutations we characterized are indicated by red asterisks (L69S; T111R/A; T232I; K243R; I245F; D255V; V260A; A285T; D287V; I293T; V310A; R376T). The *E.coli* residues that confer MinC resistance are indicated by blue asterisks (R271; E276; N280; D373; I374; L378; K380).
(TIF)

Figure S3 **CD spectroscopy of purified FtsZ proteins.** Circular dichroism spectra in the far-UV region (193–260 nm) were measured on a Jasco J-810 spectropolarimeter with a Peltier temperature control unit (Jasco Corp. Tokyo, Japan). Wild type FtsZ and mutants were measured at 5 µM in 1 mM Tris-HCl pH 8.0, 1 mM KCl, 0.02 mM EDTA and 0.2% glycerol in a 1 mm path length quartz cell at a scanning speed of 100 nm/min and by the averaging of 10 scans.
(TIF)

Figure S4 **Light scattering traces of MinC inhibition of individual FtsZ mutants.** Light scattering traces of polymerization reactions with 7 µM FtsZ and 20 µM MinC in buffer (Mes/NaOH 50 mM, MgCl2 10 mM, KCl 133 mM, DEAE-dextran 0.6 mg/mL, pH 6,5), polymerized with 1 mM GTP.
(TIF)

Figure S5 **Sedimentation assay of FtsZ^WT^ and mutants visualized by SDS-PAGE.** Reactions contained 7 µM of FtsZ in polymerization buffer (Mes/NaOH 50 mM, MgCl_2_ 10 mM, KCl 133 mM, DEAE-dextran 0.6 mg/mL, pH 6.5) with GTP 2 mM were assembled at room temperature and spun down at 100.000 rpm for 15 min using a TLA 120.1 rotor. The pellet (P) was resuspended in the same volume as the supernatant (S) and applied in a 15% polyacrylamide gel.
(TIF)

Figure S6 **Kinetics of GTP hydrolysis by mutant FtsZ.** 10 µM of purified His6-FtsZ mutants were assayed as described in Material and Methods and the concentration of inorganic phosphate measured every 2 minutes for 14 minutes. The activity was determined by calculating the slope of the resulting line.
(TIF)

Figure S7 **Determination of critical concentration of polymerization of FtsZ mutants.** Purified His6-FtsZ mutants were assayed and their enzymatic activity were determined as described in Material and Methods. The critical concentration (Cc) of each mutant FtsZ was calculated by plotting the GTPase activity in different protein concentrations and finding the intersection between the resulting line and the x axis.
(TIF)

Figure S8 **Evaluation of MinC tryptophan mutants.** A, B) Light scattering traces of polymerization reactions with 7 µM FtsZ and 20 µM MinC Y44W or F62W in Mes/NaOH 50 mM, MgCl_2_ 10 mM, KCl 133 mM, DEAE-dextran 0.6 mg/mL, pH 6,5 followed for 300s. Black line, polymerization in the absence of MinC, red line, polymerization in the presence of MinC. C, D) Fluorescence emission spectra of MinC Y44W (C) and F62W (D) in the absence (black lines) or presence (red lines) of FtsZ. MinC was at 1 µM, FtsZ at 2 µM in Tris/HCl 20 mM, KCl 100 mM, EDTA 5 mM, pH 7.5. Excitation 295 nm/emmision 320–360 nm.
(TIF)

Figure S9 **Titration of MinC Y44W with wild-type FtsZ.** Fluorescence emission spectra of 1 µM MinC Y44W in the presence of 0.5; 1; 1.5 and 2 µM FtsZ in Tris/HCl 20 mM, KCl 100 mM, EDTA 5 mM, pH 7.5.
(TIF)

Figure S10 **Effect of wild-type and K243R D287V double mutant FtsZ on MinC Y44W fluorescence.** The graph represents the mean and standard deviations of three independent experiments in which we compared the effect of 1, 2 and 3 µM FtsZ (wild-type or K243R, D287V) in the fluorescence of MinC Y44W.
(TIF)

Figure S11 **Trp fluorescence experiment showing the effect of salt on MinC-FtsZ interaction.** Wild-type FtsZ **(**2 µM) was mixed with 1 µM MinC Y44W in buffer Tris/HCl 20 mM, EDTA 5 mM, pH 7,5, in the presence of either 100 mM or 1 M KCl. Fluorescence emission at 320–360 nm was recorded.
(TIF)

Table S1 Strain and plasmid list.
(PDF)

Table S2 Oligonucleotide list.
(PDF)

Text S1 **Description of plasmid and strain construction, and supporting information references.**
(PDF)
