# Supplementary figures and images for: Genetic and Biochemical Characterization of the MinC-FtsZ Interaction in Bacillus subtilis
Source: PLoS One. 2013 Apr 5;8(4):e60690. doi: 10.1371/journal.pone.0060690 (PMC3618327; doi:10.1371/journal.pone.0060690)

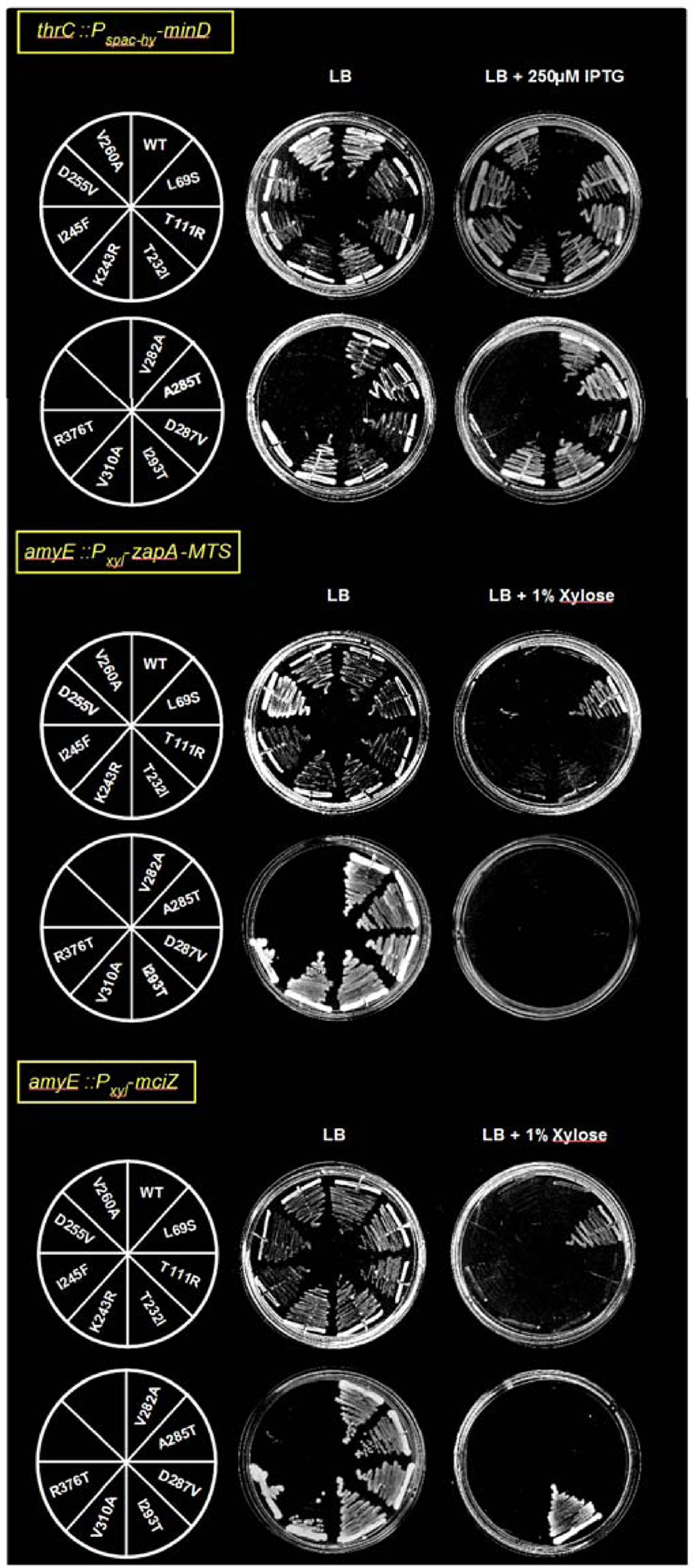

Supplement: Figure S1 — Cross-resistance of FtsZ mutants. Mutants were evaluated in three different strain backgrounds, each capable of overexpressing a different FtsZ modulator (top: MinD; middle: ZapA-MTS; bottom: MciZ). Strains were streaked onto a control plate or a plate containing inducer to promote overexpression of MinD, ZapA-MTS or MciZ. Growth was scored after overnight incubation at 37°C. (TIF) [file pone.0060690.s001.tif]

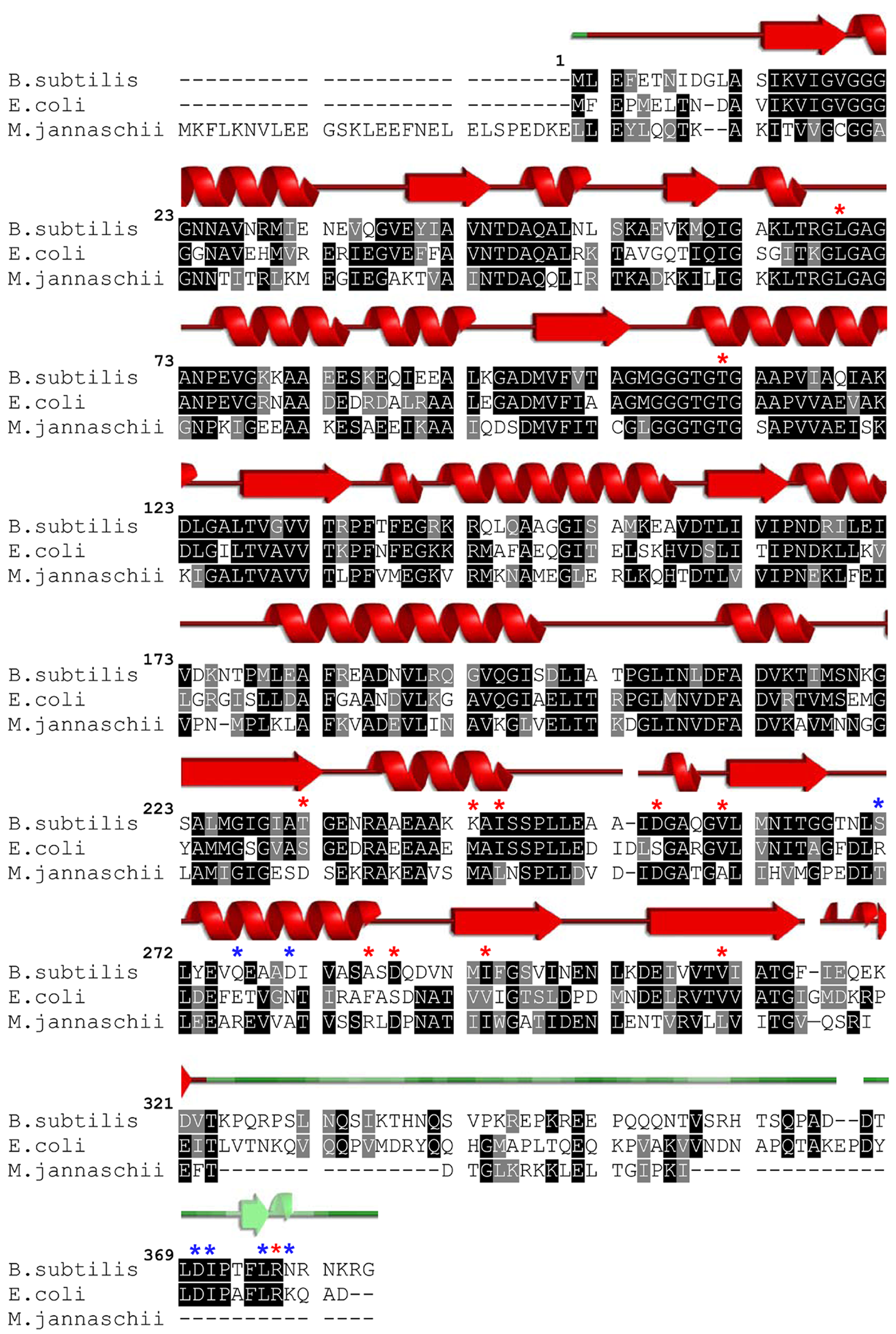

Supplement: Figure S2 — Amino acid sequence alignment of FtsZ proteins, from B. subtilis (P17865), E. coli (P0A9A6) and M. jannaschii (Q57816) using ClustalW2. Secondary structure prediction using Sequence Annotated by Structure-SAS (7) for B. subtilis FtsZ is shown above the alignment. The residues affected in the Min-resistant mutations we characterized are indicated by red asterisks (L69S; T111R/A; T232I; K243R; I245F; D255V; V260A; A285T; D287V; I293T; V310A; R376T). The E.coli residues that confer MinC resistance are indicated by blue asterisks (R271; E276; N280; D373; I374; L378; K380). (TIF) [file pone.0060690.s002.tif]

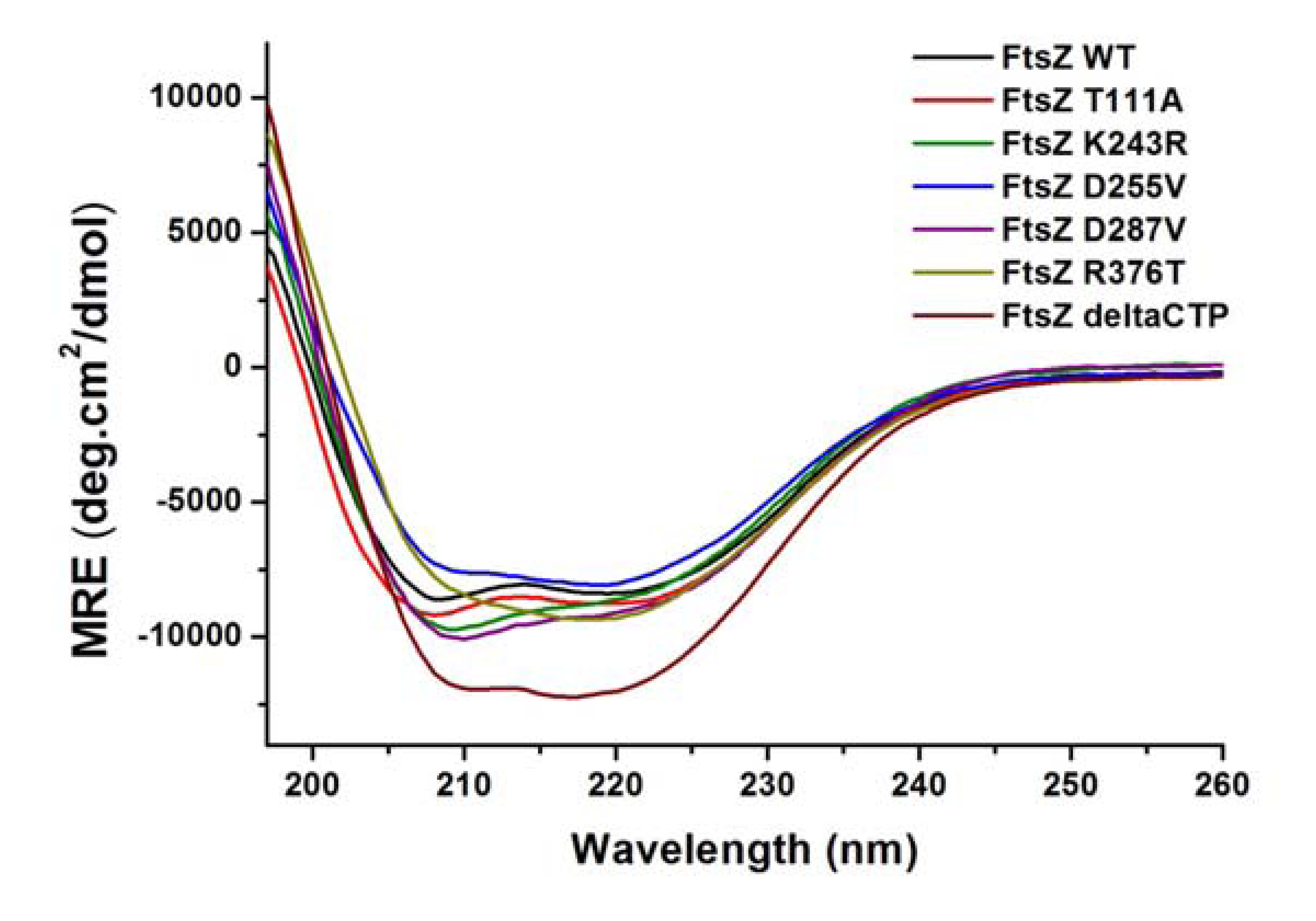

Supplement: Figure S3 — CD spectroscopy of purified FtsZ proteins. Circular dichroism spectra in the far-UV region (193–260 nm) were measured on a Jasco J-810 spectropolarimeter with a Peltier temperature control unit (Jasco Corp. Tokyo, Japan). Wild type FtsZ and mutants were measured at 5 µM in 1 mM Tris-HCl pH 8.0, 1 mM KCl, 0.02 mM EDTA and 0.2% glycerol in a 1 mm path length quartz cell at a scanning speed of 100 nm/min and by the averaging of 10 scans. (TIF) [file pone.0060690.s003.tif]

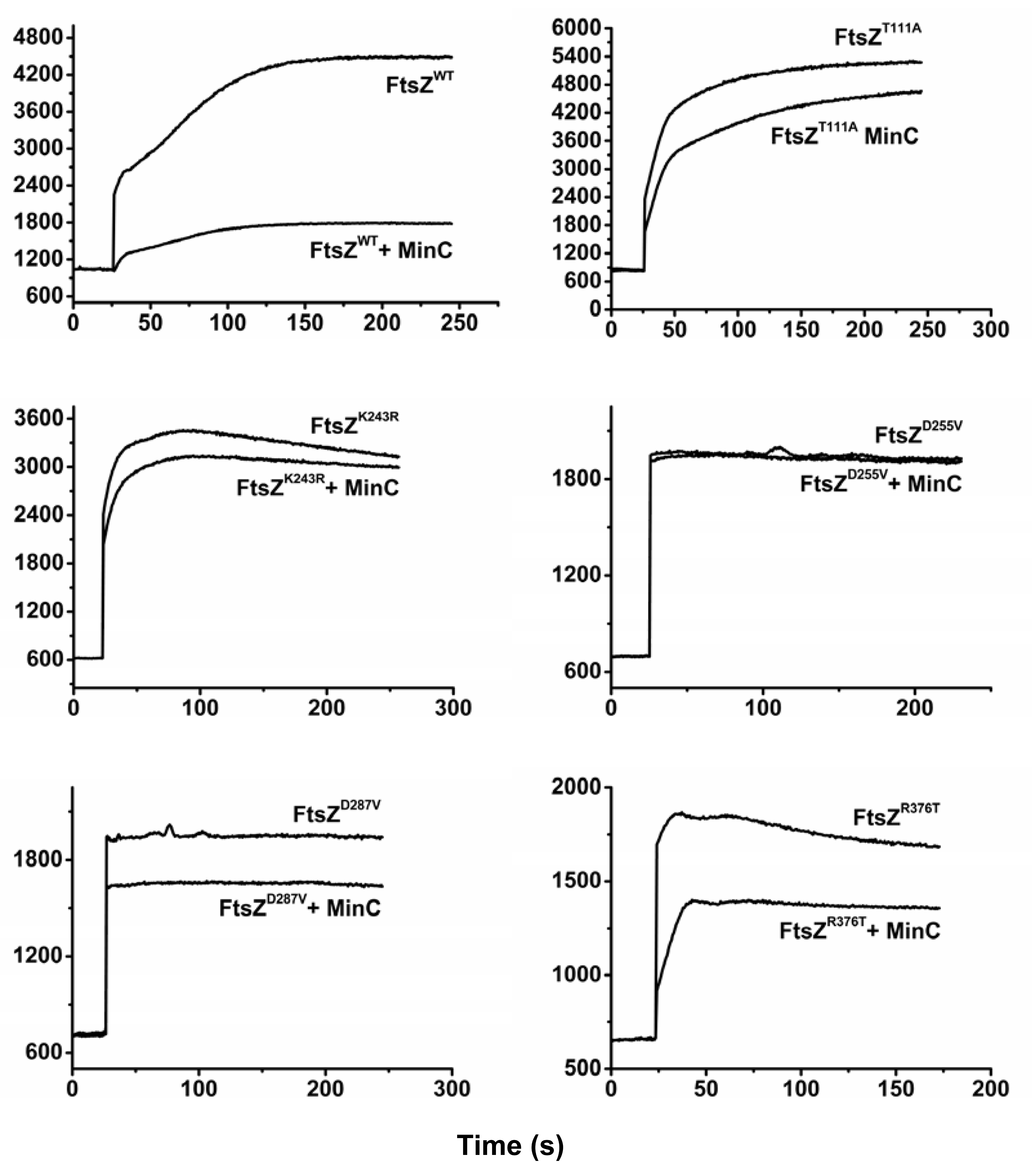

Supplement: Figure S4 — Light scattering traces of MinC inhibition of individual FtsZ mutants. Light scattering traces of polymerization reactions with 7 µM FtsZ and 20 µM MinC in buffer (Mes/NaOH 50 mM, MgCl2 10 mM, KCl 133 mM, DEAE-dextran 0.6 mg/mL, pH 6,5), polymerized with 1 mM GTP. (TIF) [file pone.0060690.s004.tif]

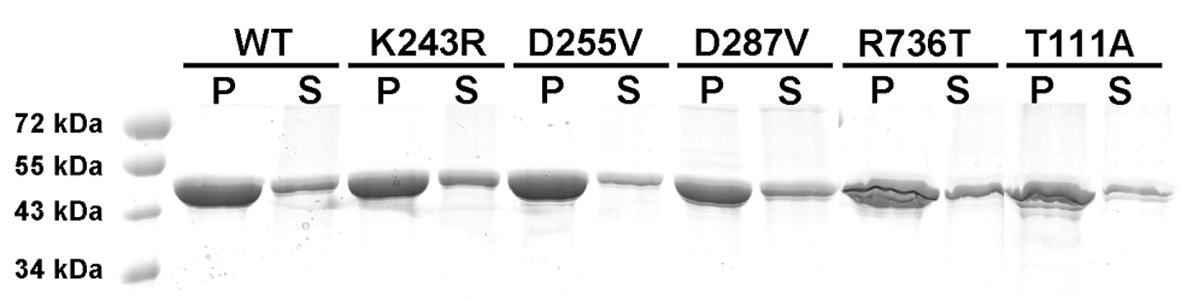

Supplement: Figure S5 — Sedimentation assay of FtsZWT and mutants visualized by SDS-PAGE. Reactions contained 7 µM of FtsZ in polymerization buffer (Mes/NaOH 50 mM, MgCl2 10 mM, KCl 133 mM, DEAE-dextran 0.6 mg/mL, pH 6.5) with GTP 2 mM were assembled at room temperature and spun down at 100.000 rpm for 15 min using a TLA 120.1 rotor. The pellet (P) was resuspended in the same volume as the supernatant (S) and applied in a 15% polyacrylamide gel. (TIF) [file pone.0060690.s005.tif]

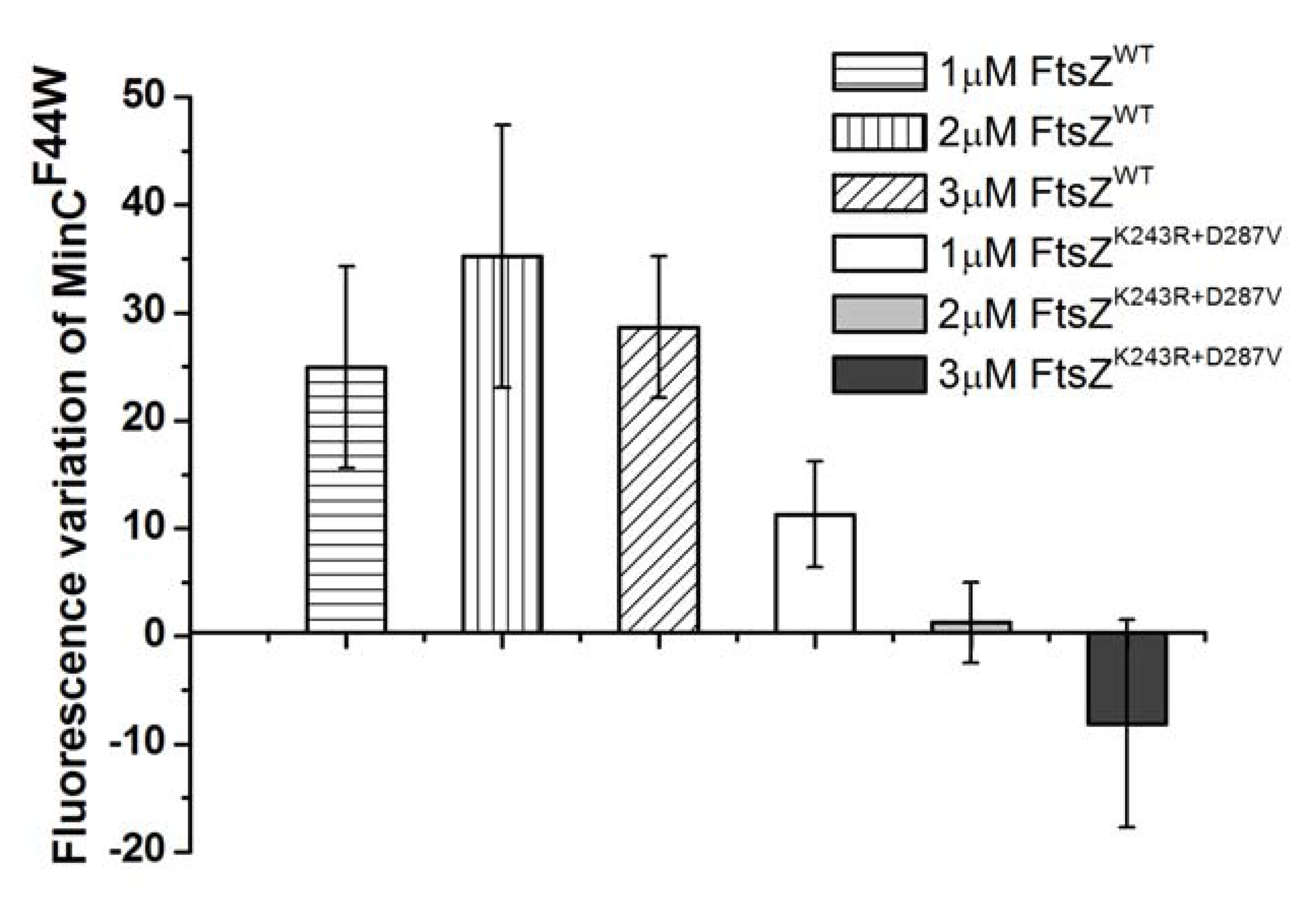

Supplement: Figure S10 — Effect of wild-type and K243R D287V double mutant FtsZ on MinC Y44W fluorescence. The graph represents the mean and standard deviations of three independent experiments in which we compared the effect of 1, 2 and 3 µM FtsZ (wild-type or K243R, D287V) in the fluorescence of MinC Y44W. (TIF) [file pone.0060690.s010.tif]

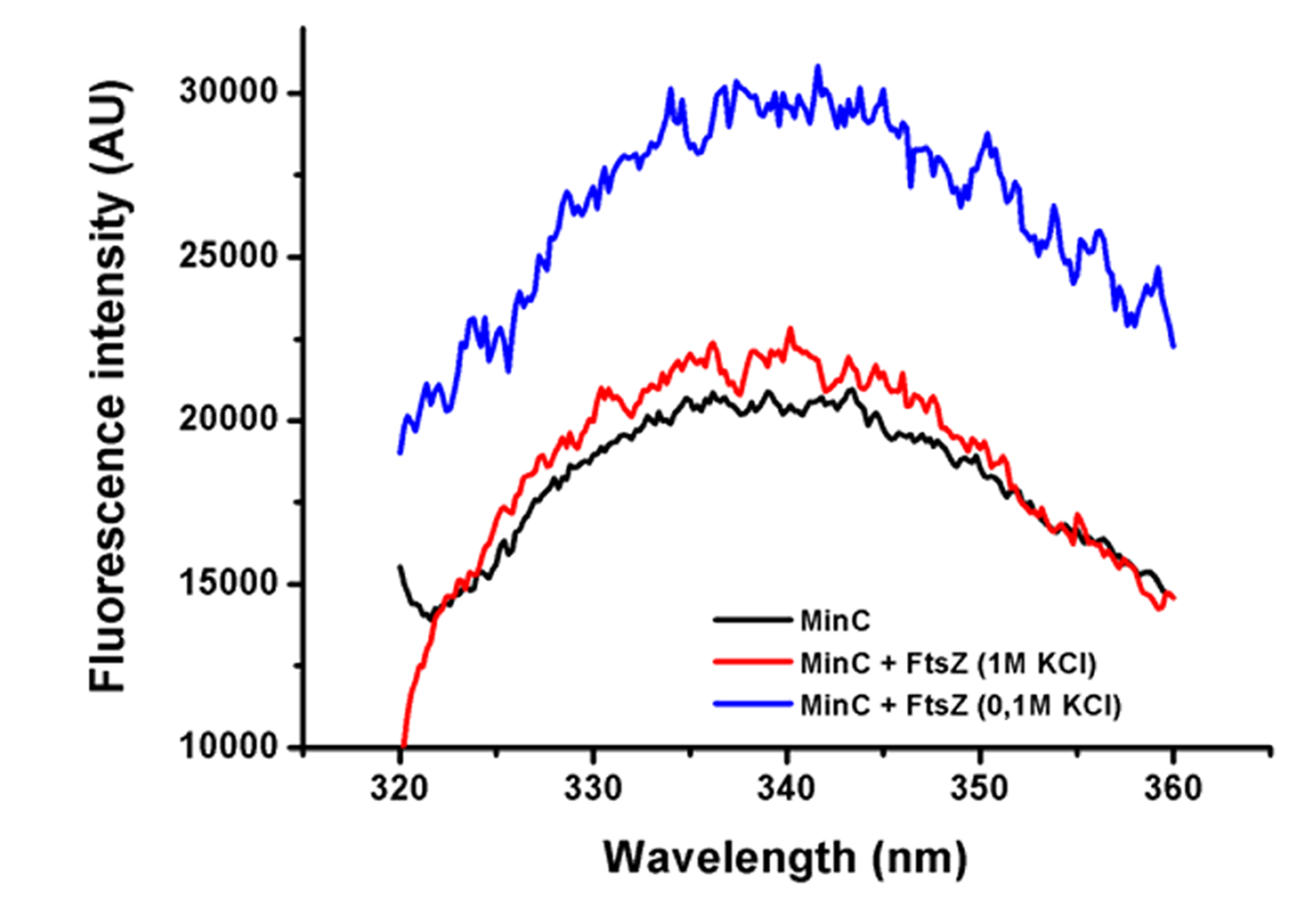

Supplement: Figure S11 — Trp fluorescence experiment showing the effect of salt on MinC-FtsZ interaction. Wild-type FtsZ (2 µM) was mixed with 1 µM MinC Y44W in buffer Tris/HCl 20 mM, EDTA 5 mM, pH 7,5, in the presence of either 100 mM or 1 M KCl. Fluorescence emission at 320–360 nm was recorded. (TIF) [file pone.0060690.s011.tif]
