# Supplementary material for: Genetic and Biochemical Characterization of the MinC-FtsZ Interaction in Bacillus subtilis
Source: PLoS One. 2013 Apr 5;8(4):e60690. doi: 10.1371/journal.pone.0060690 (PMC3618327; doi:10.1371/journal.pone.0060690)
Supplement: Table S1 — Strain and plasmid list. (PDF) [file pone.0060690.s012.pdf]

Table S1

**Bacterial strains and plasmids used in this study**

| **Strain or Plasmid** | **Genotype** | **Reference or Source** |
|---|---|---|
| *B. subtilis* | | |
| PY79 | Prototroph | (2) |
| FG247 | *thrC*::P*spac-hy-minD* (*erm*) | (3) |
| IS75 | *metB5 hisAl leuA* | D. Dubnau |
| BD3196 | *metB5 hisAl leuA roKΔ::kan* | (4) |
| AB52 | *metB5 hisAl leuA roKΔ::kan amyE::Pxyl-mciZ* | This Work |
| AB53 | *metB5 hisAl leuA roKΔ::kan amyE::Pxyl-gfp-zapA-mts* | This Work |
| AB62 | *metB5 hisAl leuA roKΔ::kan amyE::Pxyl-mciZ chrΩftsZ*$^{T111A}$ *(tet)* | This Work |
| AB70 | *thrC::Pspac-hy-minD (erm), chrΩftsZ*$^{T111A}$ *(tet)* | This Work |
| AB83 | *metB5 hisAl leuA roKΔ::kan amyE::Pxyl-gfp-zapA-mts chrΩftsZ*$^{T111A}$ *(tet)* | This Work |
| AB164 | *thrC::Pspac-hy-minD (erm) chrΩpAB10 (tet)* | This Work |
| AB165 | *thrC::Pspac-hy-minD (erm) chrΩftsZ*$^{L69S}$ *(tet)* | This Work |
| AB166 | *thrC::Pspac-hy-minD (erm) chrΩftsZ*$^{T111R}$ *(tet)* | This Work |
| AB167 | *thrC::Pspac-hy-minD (erm) chrΩftsZ*$^{T232I}$ *(tet)* | This Work |
| AB168 | *thrC::Pspac-hy-minD (erm) chrΩftsZ*$^{K243R}$ *(tet)* | This Work |
| AB169 | *thrC::Pspac-hy-minD (erm) chrΩftsZ*$^{I245F}$ *(tet)* | This Work |
| AB170 | *thrC::Pspac-hy-minD (erm) chrΩftsZ*$^{D255V}$ *(tet)* | This Work |
| AB171 | *thrC::Pspac-hy-minD (erm) chrΩftsZ*$^{V260A}$ *(tet)* | This Work |
| AB172 | *thrC::Pspac-hy-minD (erm) chrΩftsZ*$^{V282A}$ *(tet)* | This Work |
| AB173 | *thrC::Pspac-hy-minD (erm) chrΩftsZ*$^{A285T}$ *(tet)* | This Work |
| AB174 | *thrC::Pspac-hy-minD (erm) chrΩftsZ*$^{D287V}$ *(tet)* | This Work |
| AB175 | *thrC::Pspac-hy-minD (erm) chrΩftsZ*$^{I293T}$ *(tet)* | This Work |
| AB176 | *thrC::Pspac-hy-minD (erm) chrΩftsZ*$^{V310A}$ *(tet)* | This Work |
| AB177 | *thrC::Pspac-hy-minD (erm) chrΩftsZ*$^{R376T}$ *(tet)* | This Work |
| AB178 | *metB5 hisAl leuA roKΔ::kan amyE::Pxyl-mciZ (cat) chrΩpAB10 (tet)* | This Work |
| AB179 | *metB5 hisAl leuA roKΔ::kan amyE::Pxyl-mciZ (cat) chrΩftsZ*$^{L69S}$ *(tet)* | This Work |
| AB180 | *metB5 hisAl leuA roKΔ::kan amyE::Pxyl-mciZ (cat) chrΩftsZ*$^{T111R}$ *(tet)* | This Work |
| AB181 | *metB5 hisAl leuA roKΔ::kan amyE::Pxyl-mciZ (cat) chrΩftsZ*$^{T232I}$ *(tet)* | This Work |
| AB182 | *metB5 hisAl leuA roKΔ::kan amyE::Pxyl-mciZ (cat) chrΩftsZ*$^{K243R}$ *(tet)* | This Work |
| AB183 | *metB5 hisAl leuA roKΔ::kan amyE::Pxyl-mciZ (cat) chrΩftsZ*$^{I245F}$ *(tet)* | This Work |
| AB184 | *metB5 hisAl leuA roKΔ::kan amyE::Pxyl-mciZ (cat) chrΩftsZ*$^{D255V}$ *(tet)* | This Work |
| AB185 | *metB5 hisAl leuA roKΔ::kan amyE::Pxyl-mciZ (cat) chrΩftsZ*$^{V260A}$ *(tet)* | This Work |
| AB186 | *metB5 hisAl leuA roKΔ::kan amyE::Pxyl-mciZ (cat) chrΩftsZ*$^{V282A}$ *(tet)* | This Work |
| AB187 | *metB5 hisAl leuA roKΔ::kan amyE::Pxyl-mciZ (cat) chrΩftsZ*$^{A285T}$ *(tet)* | This Work |
| AB188 | *metB5 hisAl leuA roKΔ::kan amyE::Pxyl-mciZ (cat) chrΩftsZ*$^{D287V}$ *(tet)* | This Work |
| AB189 | *metB5 hisAl leuA roKΔ::kan amyE::Pxyl-mciZ (cat) chrΩftsZ*$^{I293T}$ *(tet)* | This Work |
| AB190 | *metB5 hisAl leuA roKΔ::kan amyE::Pxyl-mciZ (cat), chrΩftsZ*$^{V310A}$ *(tet)* | This Work |
| AB191 | *metB5 hisAl leuA roKΔ::kan amyE::Pxyl-mciZ (cat), chrΩftsZ*$^{R376T}$ *(tet)* | This Work |
| AB192 | *metB5 hisAl leuA roKΔ::kan amyE::Pxyl-gfp-zapA-mts (cat), chrΩpAB10 (tet)* | This Work |
| AB193 | *metB5 hisAl leuA roKΔ::kan amyE::Pxyl-gfp-zapA-mts (cat), chrΩftsZ*$^{L69S}$ *(tet)* | This Work |

| | | |
|---|---|---|
| AB194 | *metB5 hisAl leuA roKΔ::kan amyE::Pxyl-gfp-zapA-mts (cat), chrΩftsZ*$^{T111R}$ *(tet)* | This Work |
| AB195 | *metB5 hisAl leuA roKΔ::kan amyE::Pxyl-gfp-zapA-mts (cat), chrΩftsZ*$^{T232I}$ *(tet)* | This Work |
| AB196 | *metB5 hisAl leuA roKΔ::kan amyE::Pxyl-gfp-zapA-mts (cat), chrΩftsZ*$^{K243R}$ *(tet)* | This Work |
| AB197 | *metB5 hisAl leuA roKΔ::kan amyE::Pxyl-gfp-zapA-mts (cat), chrΩftsZ*$^{I245F}$ *(tet)* | This Work |
| AB198 | *metB5 hisAl leuA roKΔ::kan amyE::Pxyl-gfp-zapA-mts (cat), chrΩftsZ*$^{D255V}$ *(tet)* | This Work |
| AB199 | *metB5 hisAl leuA roKΔ::kan amyE::Pxyl-gfp-zapA-mts (cat), chrΩftsZ*$^{V260A}$ *(tet)* | This Work |
| AB200 | *metB5 hisAl leuA roKΔ::kan amyE::Pxyl-gfp-zapA-mts (cat), chrΩftsZ*$^{V282A}$ *(tet)* | This Work |
| AB201 | *metB5 hisAl leuA roKΔ::kan amyE::Pxyl-gfp-zapA-mts (cat), chrΩftsZ*$^{A285T}$ *(tet)* | This Work |
| AB202 | *metB5 hisAl leuA roKΔ::kan amyE::Pxyl-gfp-zapA-mts (cat), chrΩftsZ*$^{D287V}$ *(tet)* | This Work |
| AB203 | *metB5 hisAl leuA roKΔ::kan amyE::Pxyl-gfp-zapA-mts (cat), chrΩftsZ*$^{I293T}$ *(tet)* | This Work |
| AB204 | *metB5 hisAl leuA roKΔ::kan amyE::Pxyl-gfp-zapA-mts (cat), chrΩftsZ*$^{V310A}$ *(tet)* | This Work |
| AB205 | *metB5 hisAl leuA roKΔ::kan amyE::Pxyl-gfp-zapA-mts (cat), chrΩftsZ*$^{R376T}$ *(tet)* | This Work |
| *E. coli* | | |
| DH5α | *supE44 (lac)U169 80dlacZ M15 hsdR17 recA1 endA1 gyrA96 thi1 relA1* | |
| BL21(DE3)-RIL | *B F - ompT hsdS*(rb-mb-) *dcm - TetR gal l*(*DE3*) *endA hte* (*argU ileY leuW CamR*) | |
| *Plasmids* | | |
| pDG1515 | *bla tet* | (5) |
| pAH103 | *amyE::Pxyl-mciZ* (*cat*) integration plasmid | (6) |
| pEA18 | *amyE::Pxyl-gfp* (*cat*) integration vector | (3) |
| pFG28 | *amyE*::$P_{xyl}$-*gfp-zapA* (*cat*) integration plasmid | (3) |
| pAB10 | *ftsZ*-ATG in pDG1515 (*bla*, *tet*) integration plasmid | This Work |
| pAB20 | *ftsZ* in pET28a (*kan*) | This Work |
| pAB20-T111A | *ftsZ*$^{T111A}$ in pET28a (*kan*) | This Work |
| pAB20-K243R | *ftsZ*$^{K243R}$ in pET28a (*kan*) | This Work |
| pAB20-D287V | *ftsZ*$^{D287V}$ in pET28a (*kan*) | This Work |
| pAB20-R376T | f*tsZ*$^{R376T}$ in pET28a (*kan*) | This Work |
| pAB31 | *minC* in pET28a (*kan*) | This Work |
| pAB31-G13D | *minC*$^{G13D}$ in pET28a (*kan*) | This Work |
| pAB30 | *minC* in pET24b (*kan*) | This Work |
| pAB30-G13D | *minC*$^{G13D}$ in pET24b (*kan*) | This Work |
| pAB30-Y44W | *minC*$^{Y44W}$ in pET24b (*kan*) | This Work |
| pMG01 | $P_{tac}$-*ftsZΔC*(1-349) (*bla*) | This Work |
