# Supplementary material for: Genetic and Biochemical Characterization of the MinC-FtsZ Interaction in Bacillus subtilis
Source: PLoS One. 2013 Apr 5;8(4):e60690. doi: 10.1371/journal.pone.0060690 (PMC3618327; doi:10.1371/journal.pone.0060690)
Supplement: Table S2 — Oligonucleotide list. (PDF) [file pone.0060690.s013.pdf]

Table S2

Oligonucleotides used in this study

Name	Sequence
OL1315	5' AAGCTAGCGGCCGCGTGAAGACCAAAAAG 3'
OL1316	5' AAGGATCCGCCTCACCCAATTCAC 3'
oFG5	5' AAGCTAGCGGCCGCTTGTCTGACGGCAAA 3'
oFG16	5' AAGGATCCATCCTTTTCTTTAAGCTG 3'
oFG57	5' AAGCTAGCATGTTGGAGTTCGAAACAAAC 3'
oFG63	5' AAGGATCCTTAGCCGCGTTTATTACGGTT 3'
oFG85	5' GACGCAAAACGTGAGTAACCTCAGCAGCAGAAC 3'
oFG86	5' GTTCTGCTGCTGAGGTTACTCACGTTTTGCGTC 3'
oFG139	5' GAAAAACTGGAGCGTCAGCTTAAA 3'
oFG178	5' GCTCTAGATTGGAGTTCGAAACAAAC 3'
oFG213	5' GGTCTCAGCAGGATCGAATTCCTGTTATAAAA 3'
oFG215	5' CGGTGATCAACACACAAATTAAAAACTGGTCTGATCGCATTAAGATCTTACTCCGAA 3'
oFG298	5' GGGCGGCGGAACAGGAGCAGGTGCCGCACCGGTTATCGC 3'
oFG299	5' GCGATAACCGGTGCGGCACCTGCTCCTGTTCCGCCGCCC 3'
oFG300	5' GCAAAGAGCAGATTGAAGTAGCACTTAAAGGTGCTGACATGG 3'
oFG301	5' CCATGTCAGCACCTTTAAGTGCTACTTCAATCTGCTCTTTGC 3'
oFG302	5' GCAAAGCTGACTAGAAGATTGGGAGCAGGTGCGAATCCGG 3'
oFG303	5' CCGGATTCGCACCTGCTCCCAATCTTCTAGTCAGCTTTGC 3'
oFG308	5' CGCTTCGGCGTCTGTTCAAGACGTAAACATGATTTTCGG 3'
oFG309	5' CCGAAAATCATGTTTACGTCTTGAACAGACGCCGAAGCG 3'
oFG334	5' CTTGACATCCCGACATTCTTAACAAACCGTAATAAACGCGGC 3'
oFG335	5' GCCGCGTTTATTACGGTTTGTTAAGAATGTCGGGATGTCAAG 3'
oFG353	5' ACTAGCTAGCGAAGTTATTACGATAGCCTCACCCA 3'
oFG354	5' ATATGTAACAATAAAAGACACAAAGAATGGACTAACATTG 3'
oFG355	5' CAATGTTAGTCCATTCTTTGTGTCTTTTATTGTTACATATTGC 3'
oFG405	5' CAATGGACCGATGGAAAAGGCCAGAAAATC 3'
oFG406	5' TCCATCGGTCCATTGTTCAATTGACAGCATATTCT 3'
