# Supplementary material for: Genetic and Biochemical Characterization of the MinC-FtsZ Interaction in Bacillus subtilis
Source: PLoS One. 2013 Apr 5;8(4):e60690. doi: 10.1371/journal.pone.0060690 (PMC3618327; doi:10.1371/journal.pone.0060690)
Supplement: Text S1 — Description of plasmid and strain construction, and supporting information references. (PDF) [file pone.0060690.s014.pdf]

Plasmid Construction

pAB10 contains a copy of *ftsZ* without the ATG start codon in a Campbell integration vector. The *ftsZ* gene was amplified by PCR using oFG63 and oFG178 oligonucleotides, digested with BamHI and XbaI and cloned in pDG1515 that had been cut with the same pair of enzymes.

pAB20 was constructed cloning a copy of *ftsZ* gene amplified using oligonucleotides oFG57 and oFG63. The PCR product was digested with BamHI and NotI and cloned in pET28a plasmid that had been cut with the same enzymes. This plasmid was used to express and purify His-FtsZ used in our *in vitro* assays.

The mutated versions of pAB20 (pAB20-T111A, pAB20-K243R, pAB20-D287V and pAB20-R376T) were obtained by site-directed mutagenesis, using pAB20 as a template. The PCRs products were amplified using the oligonucleotides oFG298 and oFG299 (for pAB20-T111A), oFG330 and oFG331 (for pAB20-K243R), oFG308 and oFG309 (for pAB20-D287V), oFG334 and oFG335 (for pAB20-R376T). The pAB20 version with both K243R and D287V mutations were constructed using pAB20-D287V as a template and amplified by oFG330 and oFG331 oligonucleotides.

pAB30 contains a fusion of N-terminal His-tag and a copy of *minC*. The PCR product of *minC* was amplified using OL1315 and OL1316 oligonucleotides and cleaved it with NheI and BamHI, and cloned in the plasmid pET28a treated with the same enzymes.

pAB31 is another vector for protein expression constructed by pET24b that contains a C-terminal His-tag fused and a copy of *minC*. The *minC* was amplified by PCR using oligonucleotides OL1315 and oFG353 and cleaved it with NheI and BamHI, and cloned in the plasmid treated with the same enzymes.

The mutated versions of pAB30 (pAB30-G13D) and pAB31 (pAB31-G13D and pAB31-Y44W) were obtained by site-directed mutagenesis, using pAB30 and pAB31 as templates. The PCRs products were amplified using the oligonucleotides oFG354 and oFG355 (for pAB30-G13D and pAB31-G13D), oFG405 and oFG406 (for pAB31-Y44W).

pMG01 is a derivative of pCXZ (1) for expression of a truncated version of FtsZ missing its last 33 amino acids (FtsZΔC, 1-349). It was constructed by site directed mutagenesis of pCXZ using oligonucleotides oFG85 and oFG86.

Strain Construction

AB52 was created by transforming BD3196 with plasmid pAH103, followed by selection in chloramphenicol.

AB53 was created by transforming BD3196 with the ligation product of *zapA-mts* and pEA18, followed by selection in chloramphenicol. The *zapA* PCR product was amplified using oligonucleotides oFG5 and oFG16 and pFG28 as a template. The *mts* PCR product was obtained from amphipathic helix of *minD* gene, using oligonucleotides oFG139 and oFG215 and genomic DNA of PY79 *B. subtilis* strain as a template. The *zapA-mts* PCR product was amplified from these two independent PCR

products (*zapA* and *mts*), each one containing an overlay of 30bp in the 3' *zapA* region, using oligonucleotides oFG5 and oFG215.

AB62 was created by backcross, transforming AB52 with genomic DNA of its original *ftsZ*^{T111A} mutant strain, followed by selection in tetracycline and tested for *mciZ* over expression resistance.

AB70 was created by backcross, transforming FG247 with genomic DNA of its original *ftsZ*^{T111A} mutant strain, followed by selection in tetracycline and tested for *minD* over expression resistance.

AB83 was created by backcross, transforming AB53 with genomic DNA of its original *ftsZ*^{T111A} mutant strain, followed by selection in tetracycline and tested tested for *zapA-mts* over expression resistance.

AB164 to AB177 were created by backcross, transforming FG247 with genomic DNA of each original screen mutant strain, followed by selection in tetracycline and tested for *minD* over expression resistance.

AB178 to AB191 were created by backcross, transforming AB52 with genomic DNA of each mutant strain, followed by selection in tetracycline and tested for *mciZ* over expression resistance.

AB192 to AB205 were created by backcross, transforming AB53 with genomic DNA of each mutant strain, followed by selection in tetracycline and tested for *zapA-mts* over expression resistance.

References

1. Wang X, Lutkenhaus J (1993) The FtsZ protein of Bacillus subtilis is localized at the division site and has GTPase activity that is dependent upon FtsZ concentration. Mol. Microbiol. 9, 435-442
2. Youngman PJ, Perkins JB, Losick R (1983) Genetic transposition and insertional mutagenesis in Bacillus subtilis with Streptococcus faecalis transposon Tn917. Proc Natl Acad Sci U S A. 80, 2305-2309.
3. Gueiros-Filho FJ, Losick R (2002) A widely conserved bacterial cell division protein that promotes assembly of the tubulin-like protein FtsZ. Genes Dev 16, 2544-2556
4. Albano M, Smits WK, Ho LT, Kraigher B, Mandic-Mulec I, et al. (2005) The Rok protein of Bacillus subtilis represses genes for cell surface and extracellular functions. J. Bacteriol. 187, 2010-2019
5. Guerout-Fleury AM, Shazand K, Frandsen N, Stragier P (1995) Antibiotic-resistance cassettes for Bacillus subtilis. Gene 167, 335-336
6. Handler AA, Lim JE, Losick R (2008) Peptide inhibitor of cytokinesis during sporulation in Bacillus subtilis. Mol Microbiol 68, 588-599
7. Milburn D, Laskowski RA, Thornton JM (1998) Sequences annotated by structure: a tool to facilitate the use of structural information in sequence analysis. Protein. Eng. 11, 855-859
8. Oliva MA, Trambaiolo D, Lowe J (2007) Structural insights into the conformational variability of FtsZ. J Mol Biol 373, 1229-1242
